# Supplementary material for: Genome structure-based Juglandaceae phylogenies contradict alignment-based phylogenies and substitution rates vary with DNA repair genes
Source: Nat Commun. 2023 Feb 4;14:617. doi: 10.1038/s41467-023-36247-z (PMC9899254; doi:10.1038/s41467-023-36247-z)
Supplement: Supplementary file 1 — Supplementary Information [file 41467_2023_36247_MOESM1_ESM.pdf]

Genome structure-based Juglandaceae phylogenies contradict alignment-based phylogenies and substitution rates vary with DNA repair genes

Ding *et al.*

Supplementary Note 1. Subgenome assignment based on intraspecific collinear blocks

Subgenome assignment was performed for each pair of intraspecific collinear blocks based on their numbers of retained ancestral genes[1-3]. We used reciprocal best hits (RBHs) between *Quercus lobata*[4] and *Rhoiptelea chiliantha* to obtain a data set of ancestral genes for *R. chiliantha*, and similarly for *Carya illinoinensis*[5]*, Engelhardia roxburghiana*, *Juglans regia*[6], *J. mandshurica*[7]*, J. microcarpa*[6] and *Platycarya strobilacea*[8], *Pterocarya stenoptera*[7]. To remove the complications introduced by the more ancient core eudicot *γ*-WGT, we retained only median $K_s < 1$ of collinear blocks for subgenome assignment. Each intraspecific collinear block was assigned to the two subgenomes based-on their number of retained ancestral genes. We denoted the chromosomal region with more genes as 'dominant' and that with fewer genes as 'recessive'.

Supplementary Note 2. Sequence processing and genome assemblies

For *Rhoiptelea chiliantha* and *Engelhardia roxburghiana*, PacBio long reads were corrected for errors and preassembled with falcon-kit v1.0[9] with parameters 'falcon_sense_option = --output_multi --min_idt 0.70 --min_cov 3 --max_n_read 300 --n_core 20'. The genome was polished with Arrow (SMRTLink, v5.1.0) and checked for misassembly using default parameters. To ensure the accuracy of the genome assembly, we used the BWA-mem algorithm in BWA v.0.7.15[10] with default settings to map the Illumina 150 bp paired-end clean reads to the preassembled genome. Pilon v1.22[11] was used to correct the preassembly result. Effective and high-quality Hi-C sequencing data were aligned to the draft genome with HiCUP[12] to get BAM file. The pruned BAM file was input for the ALLHIC[13] pipeline (pruning, partitioning, rescue, optimization, and building) for the chromosome-level genome assembly. Juicebox[14] was used to manually correct the chromosome-level assembly result. We used the Benchmarking Universal Single-Copy Orthologs (BUSCO) v3.0.2[15] to identify universal single copy orthologs (USCOs) in the assembly genome as a measure of completeness of the genome with the plant dataset (embryophyta_odb9).

Supplementary Note 3. Gene prediction and functional annotation of protein coding genes

For *R. chiliantha*, we used an *ab initio* search and homology alignment to identify the whole genome repeats. We extracted tandem repeats using TRF[16] with *ab initio* prediction and used homolog prediction common in the Repbase [17] database using RepeatMasker[18] and its in-house scripts (RepeatProteinMask) with default parameters to extract repeat regions. *Ab initio* prediction with default parameters in LTR_FINDER[19], RepeatScout[20], and RepeatModeler[20] were used to build *de novo* a database of repetitive elements, and all repeat sequences with lengths >100 bp and gap "N" less than 5% constituted the library of raw transposable elements (TEs). Combination of Repbase and *de novo* TE library was processed by UCLUST[21] to yield a nonredundant library, which was supplied to RepeatMasker to identify DNA-level repeats.

We used homology information, *ab initio* prediction, and RNA-seq to perform gene annotation for the *R. chiliantha* genome. For homology-based prediction, protein sequences of seven Fagales species—*Betula pendula*[22], *Carya cathayensis*[23], *Juglans regia*[6], *Myrica rubra*[24], *Pterocarya stenoptera*[7], *Quercus lobata* Version 3.0[4], and *Ostrya rehderiana*[25] were mapped to the repeat-masked genome with TblastN with an e cutoff of 10^{-5}. The matching proteins were aligned with homologous genome sequences for accurate spliced alignment with GeneWise v2.4.1[26], which was used to predict the gene structure of each protein region. For gene prediction based on *ab initio* prediction, we used Augustus v.3.2.3, Geneid v.1.4[27], Genescan v.1.0[28], GlimmerHMM v.3.04[29], and SNAP (2013-11-29)[30] in our automated gene prediction pipeline. For RNA-seq data, transcriptome read assemblies were generated with Trinity v.2.1.1[30] for genome annotation. To optimize genome annotation, RNA-seq reads from different tissues were aligned with the genome by Hisat v.2.1.0[31] with default parameters. The alignment results were then used as input for Stringtie v.1.3.3[32] with default parameters for genome-based transcript assembly. We generated a nonredundant reference gene set by merging genes predicted by these three methods with EvidenceModeler v.1.1.1[33] using PASA (Program to Assemble Spliced Alignment) [33] terminal exon support and including masked transposable elements as input for gene prediction. The annotation process for *E. roxburghiana* was the same as that used for *R. chiliantha*.

After genome annotation, we used the Kyoto Encyclopedia of Genes and Genomes (KEGG) Automatic Annotation Server (https://www.genome.jp/tools/kaas/) to perform KEGG Orthology (KO) annotation for seven species, *C. illinoinensis, E. roxburghiana*, *J. mandshurica, J. microcarpa, J. regia*, *P. strobilacea* and *R. chiliantha*. *Juglans regia*, *Quercus suber*, and the more distant *Vitis vinifera* and *Cucumis sativus* were used as references in the bidirectional best-hit method to assign orthologs and obtain KO ID. We used KEGG Ontology (ko03400) to obtain the gene related to DNA repair and recombination.

Supplementary Note 4. Selecting gene families to detect whether *R. chiliantha* shares a WGD with other Juglandaceae species

We used OrthoFinder v2.4.0[34] to identify gene families of seven Juglandaceae species (*Carya illinoinensis*[5], *Engelhardia roxburghiana*, *Juglans regia*[6], *J. microcarpa*[6], *J. mandshurica*[7], *Platycarya strobilacea*[8], *Rhoiptelea chiliantha*) and *Quercus lobata*[4]. The gene families having at least two gene copies in *R. chiliantha* or the other Juglandaceae species (*C. illinoinensis*, *E. roxburghiana*, *J. mandshurica*, *J. microcarpa*, *J. regia*, *P. strobilacea*) and having at least one gene copy in *Q. lobata* were retained in a preliminary selection. We obtained three types of gene families: (i) families in which both *R. chiliantha* and the other Juglandaceae have two gene copies; (ii) families in which *R. chiliantha* has two copies and the other species have only one; (iii) families in which *R. chiliantha* has only one copy and the other species have two copies. To ensure the homology of genes, we used MCScanX[35] to detect

intraspecific collinear blocks in the seven species of Juglandaceae and interspecific collinear blocks between *R. chiliantha* and each of the six other species and calculated the K_s values of collinear gene pairs using KaKs_Calculator 2.0[36]. To remove the complications introduced by the more ancient core eudicot *γ*-WGT, we retained only median $K_s < 1$ of collinear blocks for downstream analysis. Finally, in the first type of gene families, if two gene copies of *R. chiliantha* and the other Juglandaceae appear in the intraspecific collinear block and also in the interspecific collinear block, they were retained for analysis. For the other two types of gene families, we used the same approach to filtering them. After these filtering steps, the remained gene families were used to construct phylogenetic trees with IQ-TREE v2.1.2[37]. We calculated the frequency of each topology based on phylogenetic trees with supporting value over 80. The detailed workflow is shown in Supplementary Figure 27.

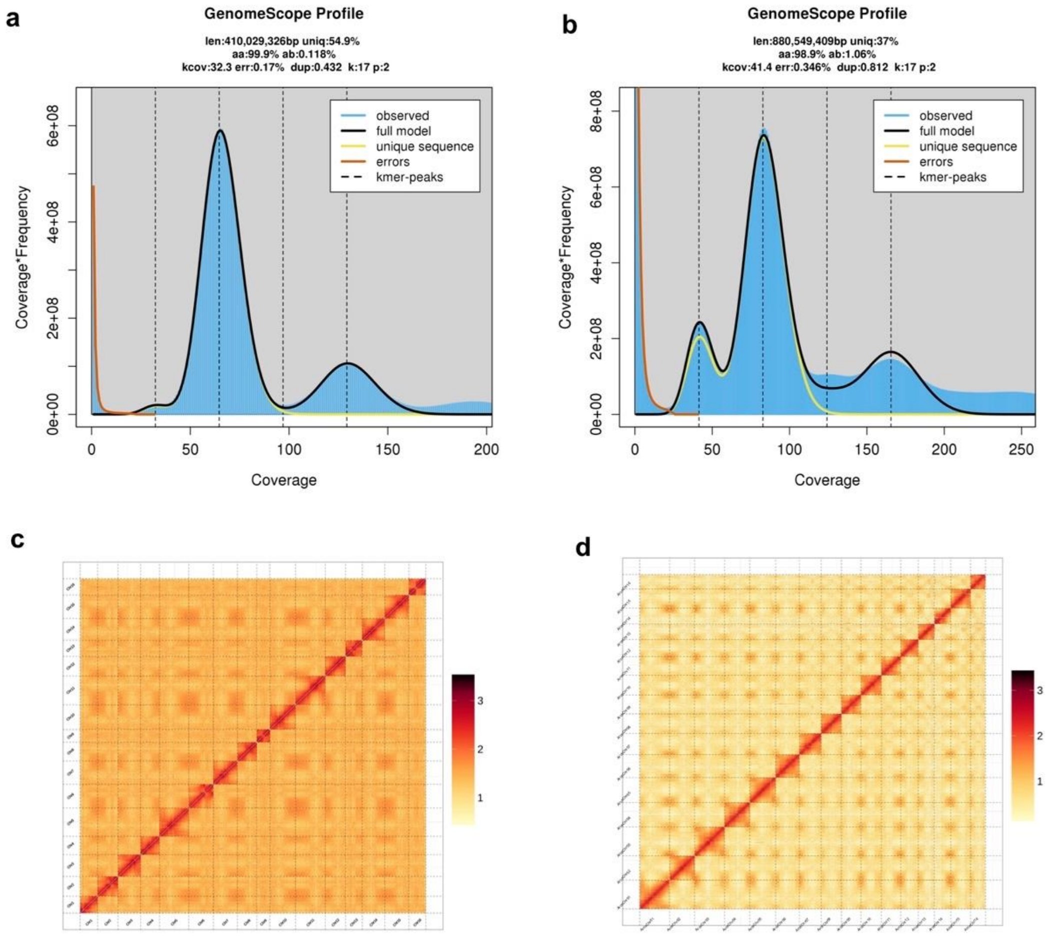

Supplementary Figure 1. GenomeScope plot and the Hi-C assisted assembly. GenomeScope plots for **(a)** *Rhoiptelea chiliantha* and **(b)** *Engelhardia roxburghiana.* **c,** The Hi-C assisted assembly of *R. chiliantha* 16 pseudo-chromosomes. Heatmap showing Hi-C chromosomal interactions. Darker red colour indicates higher contact probability. **d,** The Hi-C assisted assembly of *E. roxburghiana* 16 pseudo-chromosomes. Heatmap showing Hi-C chromosomal interactions. Darker red colour indicates higher contact probability.

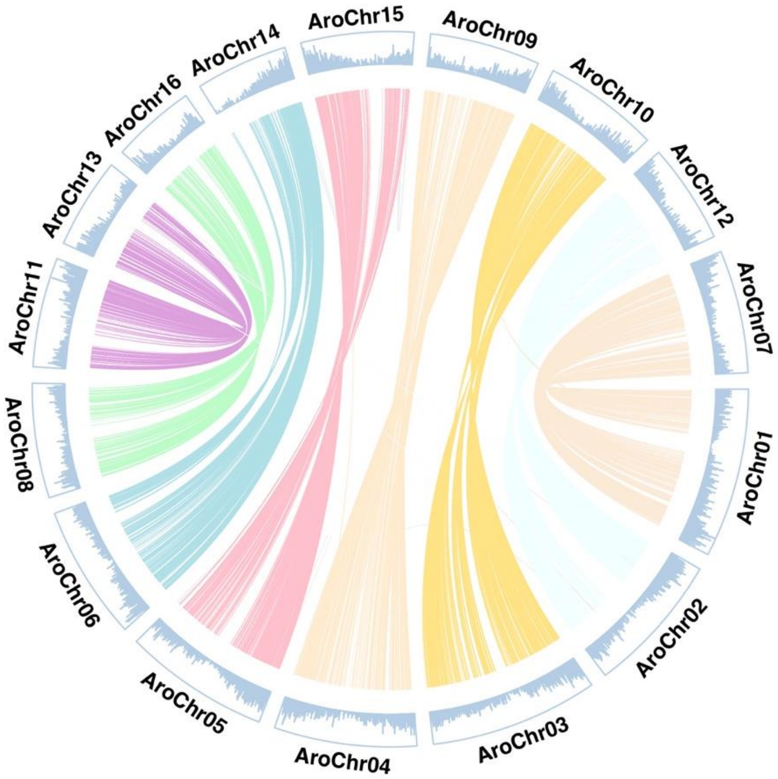

Supplementary Figure 2. A circular plot of *Engelhardia roxburghiana* homoeologous chromosomes. The reciprocal-best-hits homologous gene pairs were used in MCScanX. Central lines connect collinear blocks across chromosomes, on which gene density is shown. Different colours represent different homoeologous chromosome pairs derived from the most recent shared WGD event.

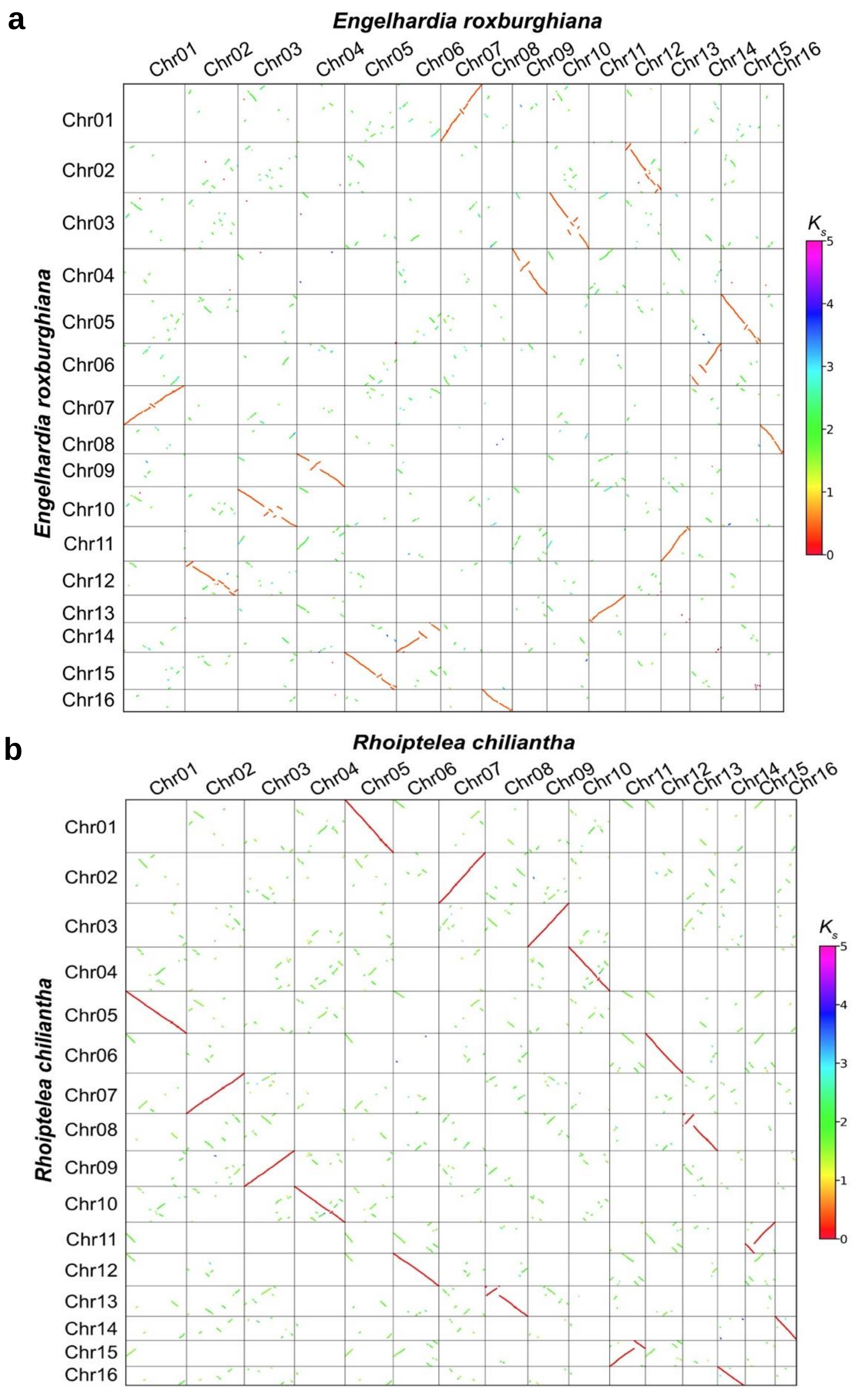

Supplementary Figure 3. Dot plot showing the syntenic relationship of homoeologous chromosomes in (a) *E. roxburghiana* and (b) *R. chiliantha*. Synteny patterns were coloured by the median K_s value in each intraspecific collinear block.

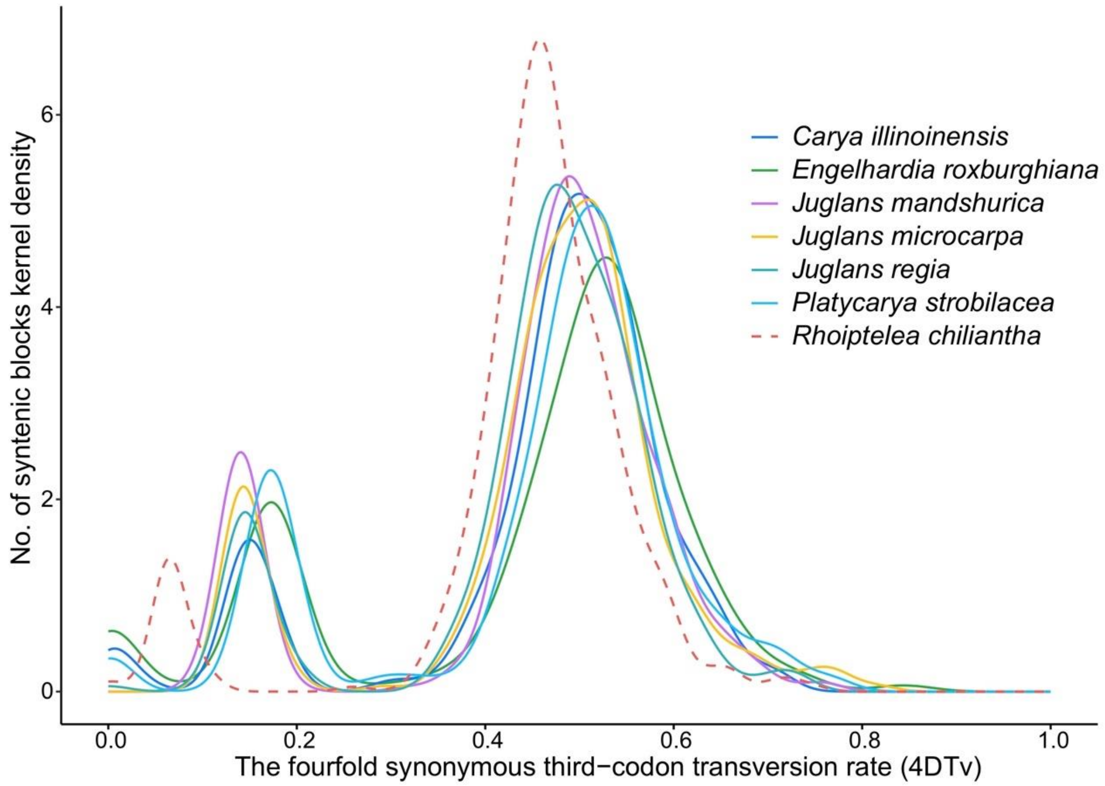

Supplementary Figure 4. The distribution of the fourfold synonymous third-codon transversion rate (4DTv) between collinear gene pairs for seven species, *C. illinoinensis, E. roxburghiana*, *J. mandshurica, J. microcarpa, J. regia*, *P. strobilacea* and *R. chiliantha*.

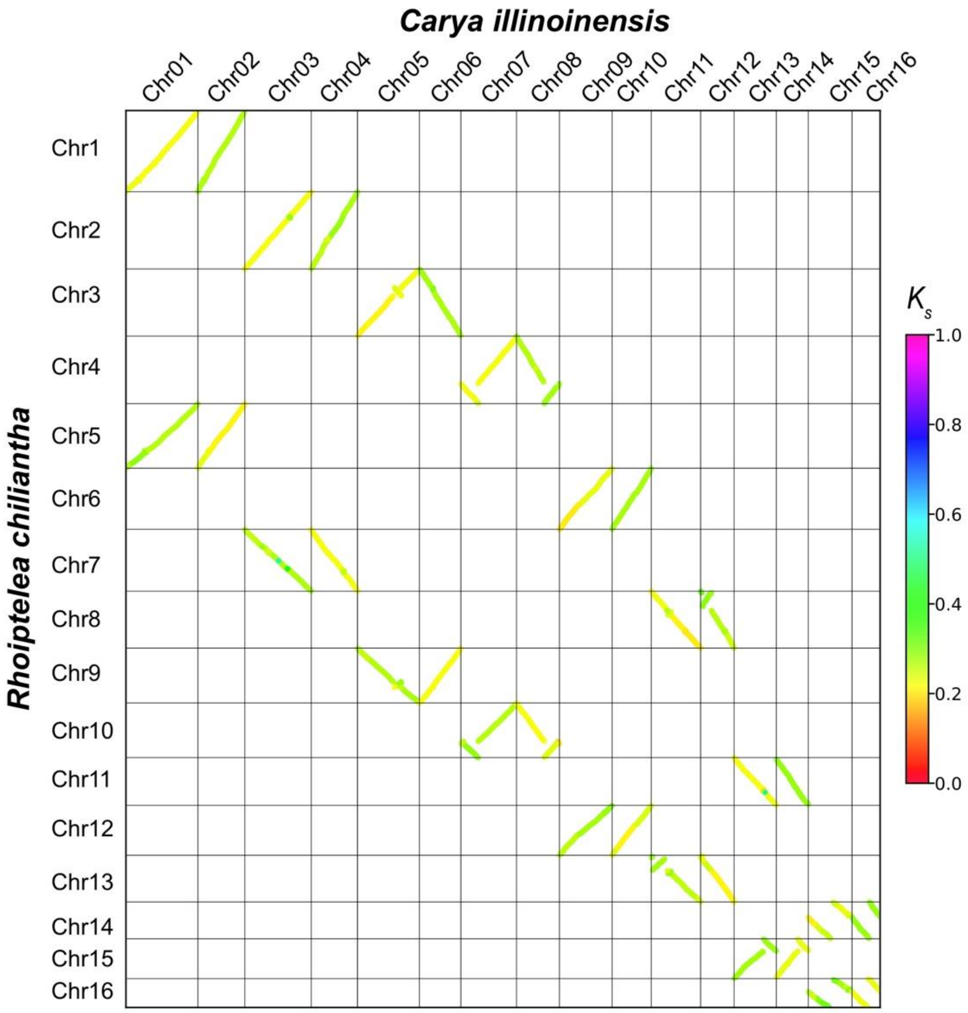

Supplementary Figure 5. Dot plot showing homologous chromosomes between *R. chiliantha* and *C. illinoinensis*. Synteny patterns visualized by dot plots coloured by the median K_s value on each collinear block.

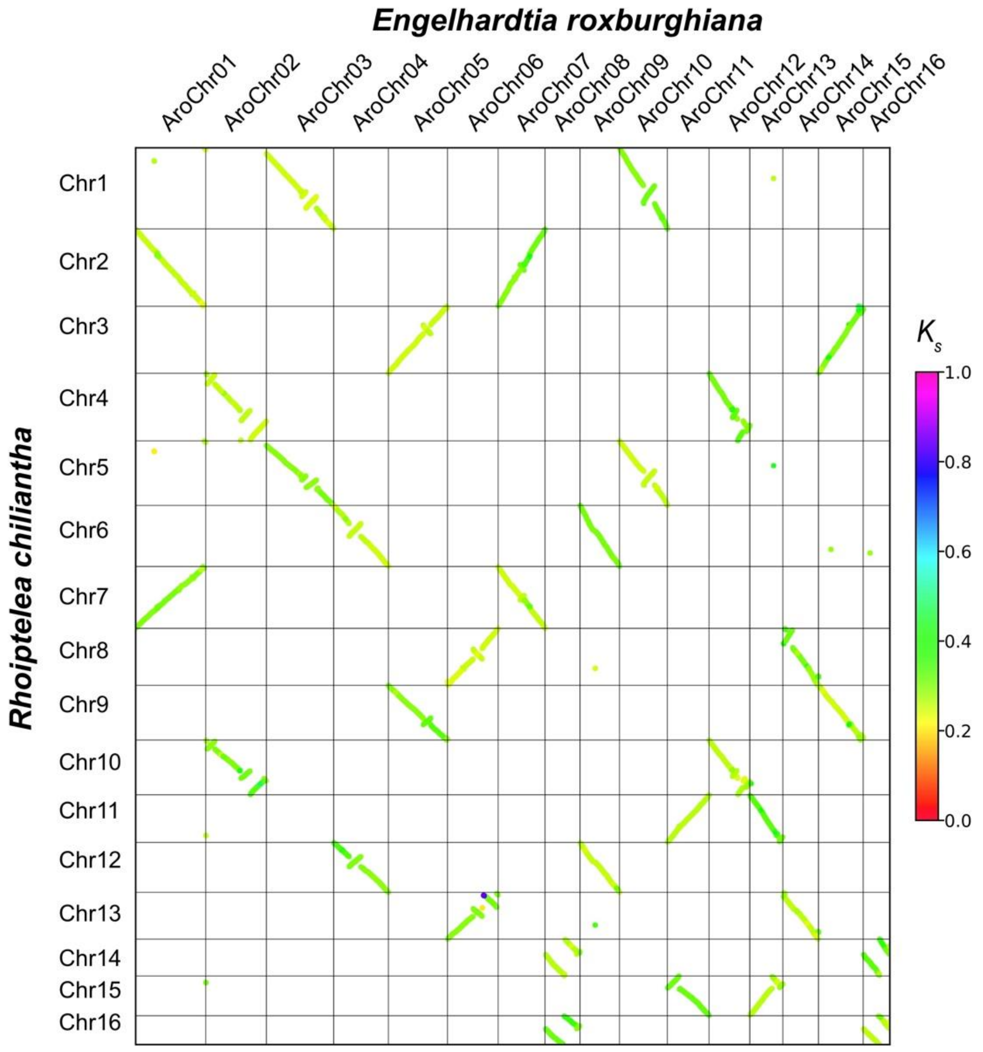

Supplementary Figure 6. Dot plot showing homologous chromosomes between *R. chiliantha* and *E. roxburghiana*. Synteny patterns visualized by dot plots coloured by the median K_s value on each collinear block.

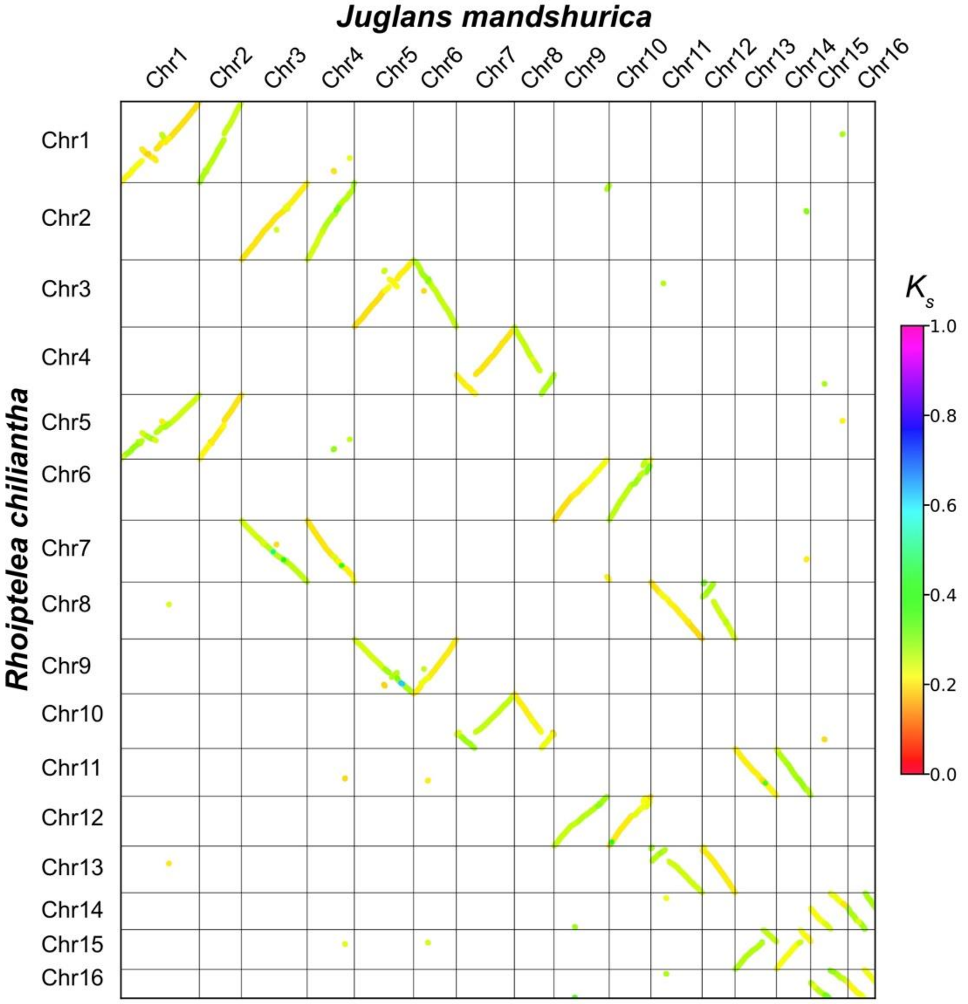

Supplementary Figure 7. Dot plot showing homologous chromosomes between *R. chiliantha* and *J. mandshurica*. Synteny patterns visualized by dot plots coloured by the median K_s value on each collinear block.

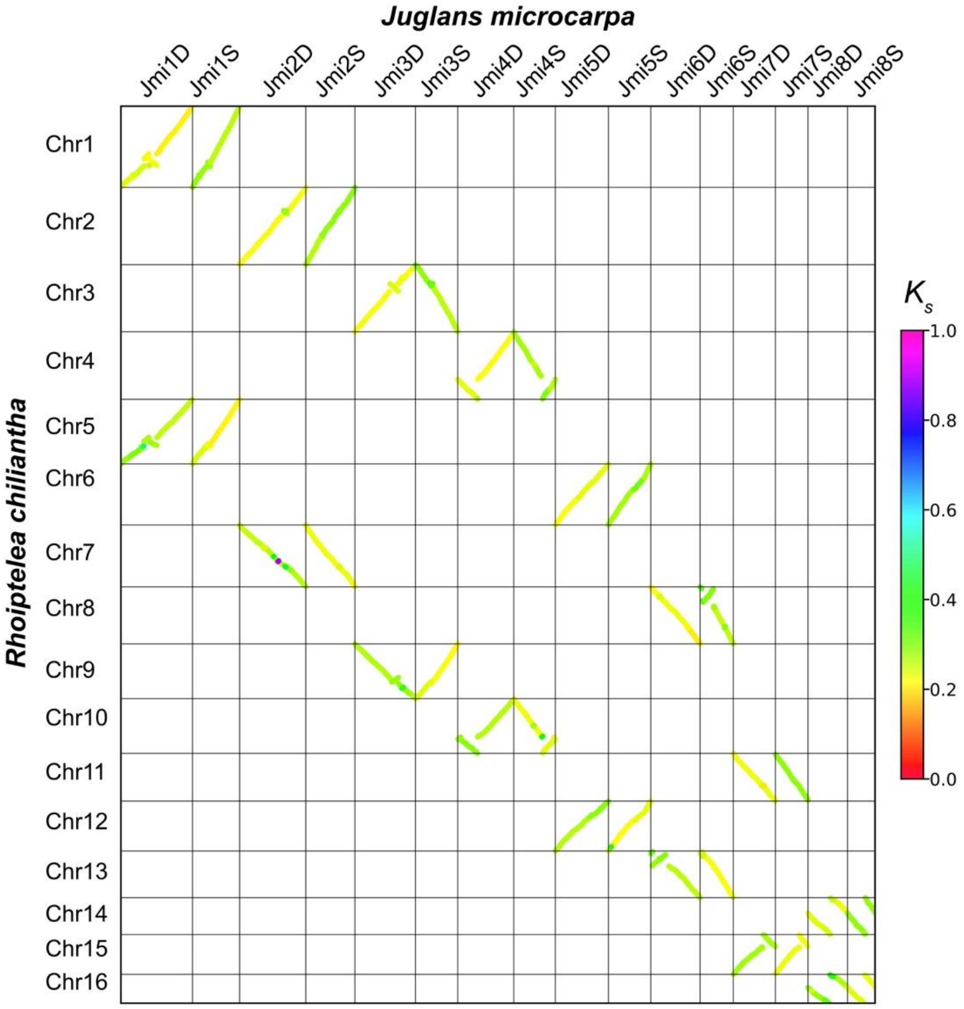

Supplementary Figure 8. Dot plot showing homologous chromosomes between *R. chiliantha* and *J. microcarpa*. Synteny patterns visualized by dot plots coloured by the median K_s value on each collinear block.

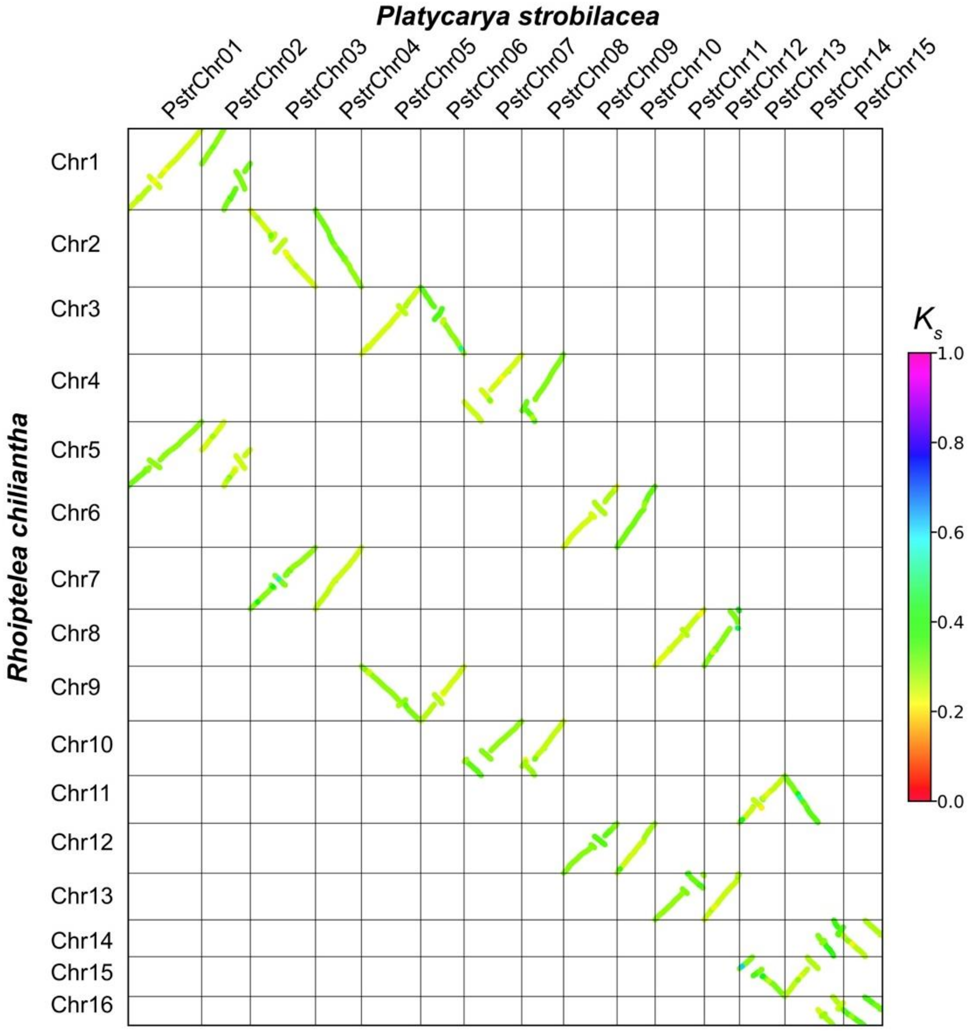

Supplementary Figure 9. Dot plot showing homologous chromosomes between *R. chiliantha* and *P. strobilacea*. Synteny patterns visualized by dot plots coloured by the median Ks value on each collinear block.

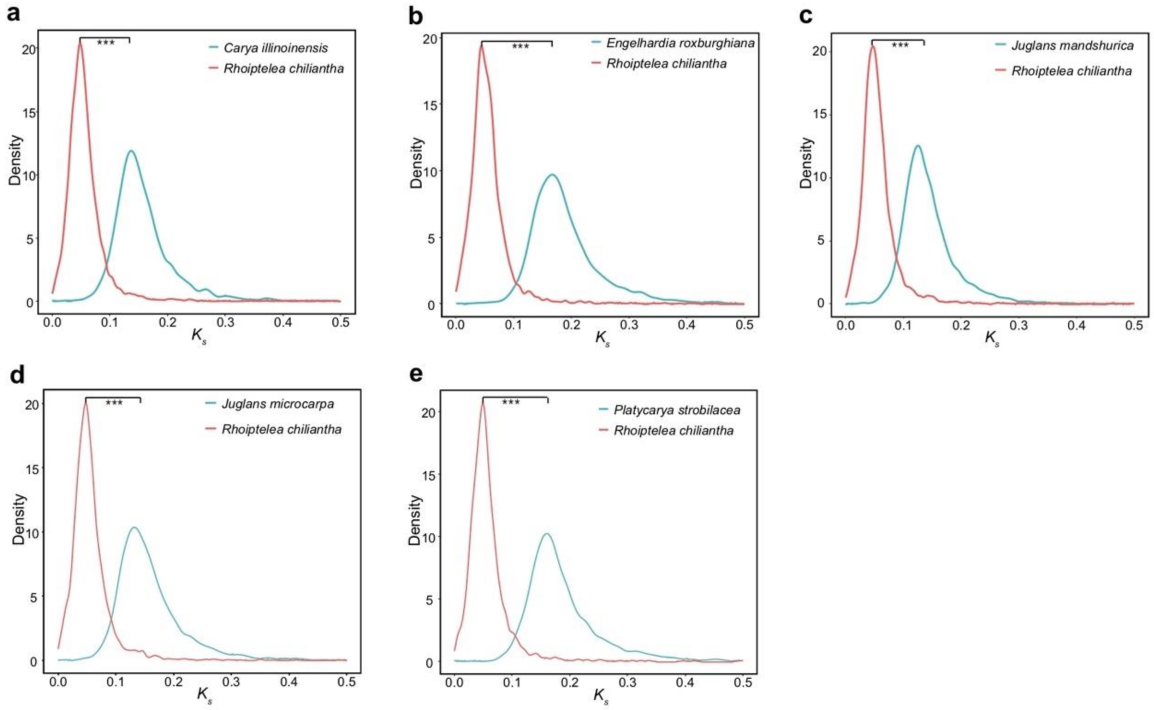

Supplementary Figure 10. *Rhoiptelea chiliantha* evolves at a markedly slower molecular rate compared to other Juglandaceae species. (a)-(e) The K_s distributions for each pair of *R. chiliantha* (red) and the another Juglandaceae species (blue). K_s is calculated as the expected number of substitutions per synonymous site between the focal species and its common ancestor with the other species. The two tailed two sample Wilcoxon rank-sum test (Mann-Whitney test) was performed on the K_s distribution of *R. chiliantha* and other species. The Juglandaceae species considered in each panel are: **(a)** *C. illinoinensis* **(b)** *E. roxburghiana* **(c)** *J. mandshurica* **(d)** *J. microcarpa* **(e)** *P. strobilacea*. *** $P < 0.001$.

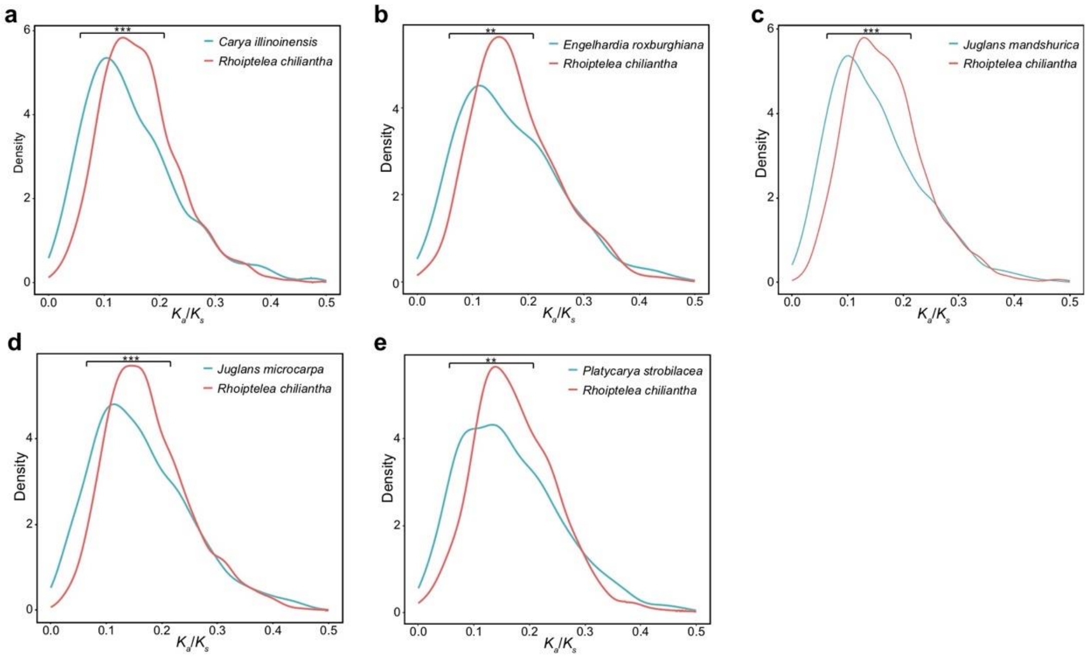

Supplementary Figure 11. The pairwise non-synonymous/synonymous mutation ratio (K_a/K_s) distribution between *R. chiliantha* (red) and the other Juglandaceae species (blue). K_a/K_s is calculated between the focal species and its most recent common ancestor with the other species. The two tailed two sample Wilcoxon rank-sum test (Mann-Whitney test) was performed on the K_s distribution of *R. chiliantha* and other species Wilcoxon rank-sum test (Mann-Whitney test) was performed for each pairwise K_a/K_s distribution after the six species divergence from the ancestor of Juglandaceae. The Juglandaceae species pairs considered in the five panel are: **(a)** *C. illinoinensis* **(b)** *E. roxburghiana* **(c)** *J. mandshurica* **(d)** *J. microcarpa* **(e)** *P. strobilacea*. ** $P< 0.01$, *** $P< 0.001$.

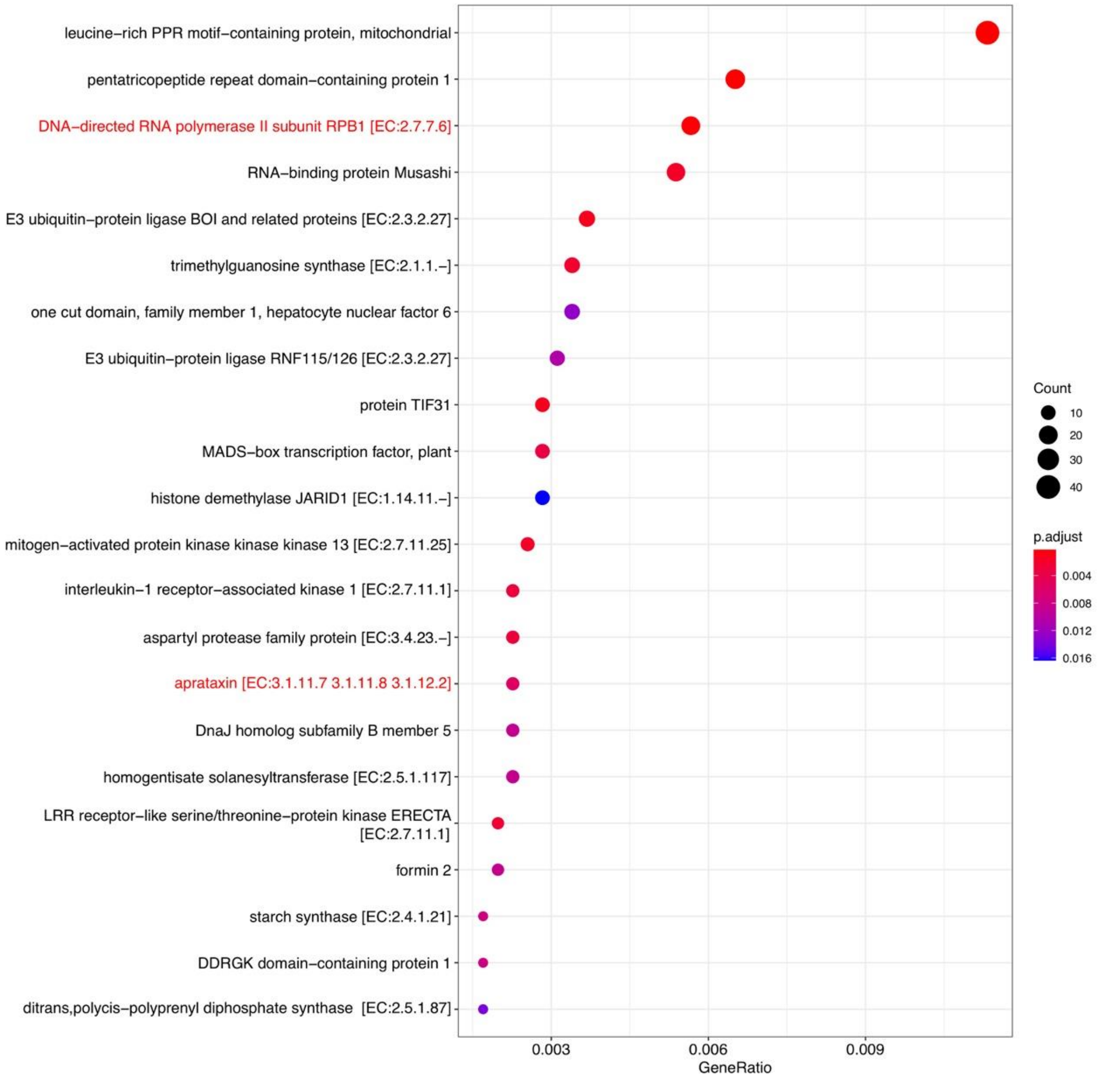

Supplementary Figure 12. KEGG enrichment analysis for the genes that have two copies in *R. chiliantha* from the juglandoid WGD, while other Juglandaceae species had at most one copy. KEGG enrichment analysis use one-sided Fisher's exact test with a *P*-value threshold of 0.05. The red functions are involved in transcription-coupled repair (RPB1) and base excision repair (Aprataxin).

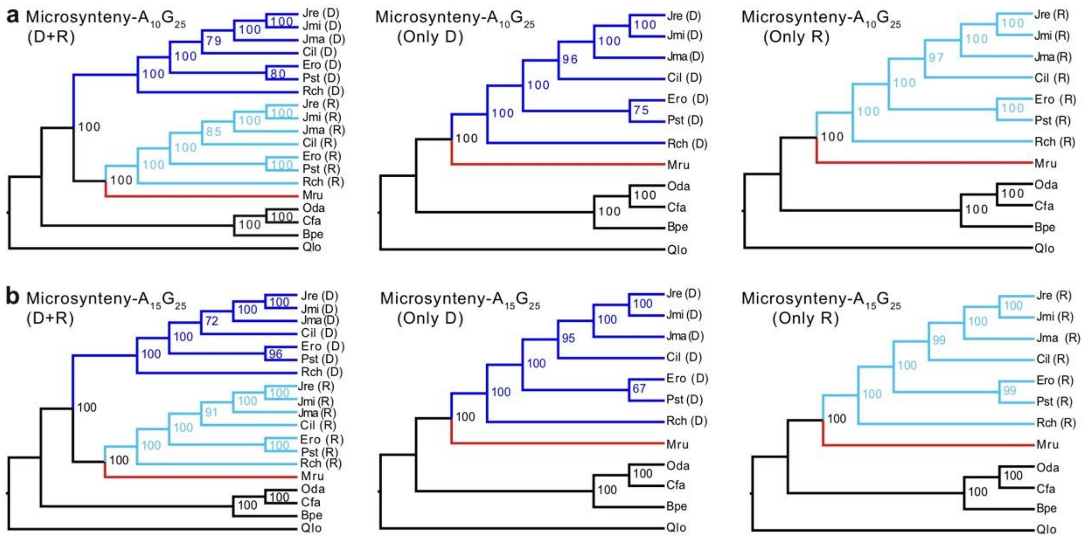

Supplementary Figure 13. Phylogenetic trees of Juglandaceae obtained from whole-genome microsynteny [Syn-MRL]. The subgenomes assigned by homoeologous chromosomes (dominant and recessive subgenomes of the seven Juglandaceae species and the five outgroups; dominant subgenome (D); recessive subgenome (R)). The Juglandaceae species are *Carya illinoinensis* (Cil), *Engelhardia roxburghiana* (Ero), *Juglans mandshurica* (Jma), *Juglans microcarpa* (Jmi), *Juglans regia* (Jre), *Platycarya strobilacea* (Pst), *Rhoiptelea chiliantha* (Rch) and the outgroups are *Betula pendula* (Bpe), *Carpinus fangiana* (Cfa), *Myrica rubra* (Mru), *Ostryopsis davidiana* (Oda), *Quercus lobata* (Qlo). The panel shows phylogenies inferred by Syn-MRL under two different parameter settings, $A_{10}G_{25}$ (A: the minimum number of anchor pairs required to call a collinear block, G: maximum number of intervening genes between two (adjacent) anchor pairs in collinear blocks) (**a**), and $A_{15}G_{25}$ (**b**), including both dominant and recessive subgenomes, only dominant subgenomes, or only recessive subgenomes. Ultrafast bootstrap (UFBoot) support (%) is shown for each node in panels.

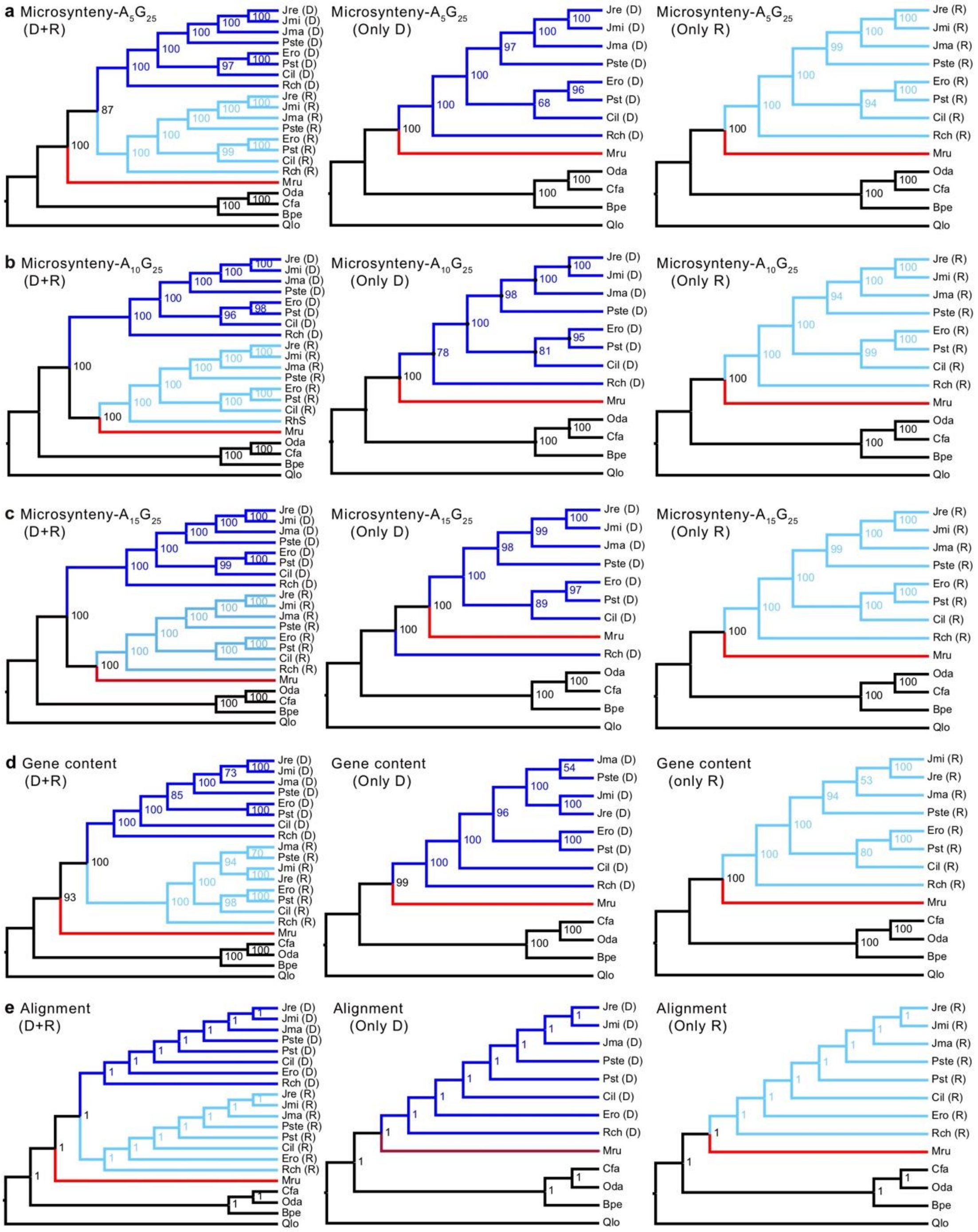

Supplementary Figure 14. Phylogenetic trees of Juglandaceae obtained from whole-genome microsynteny [Syn-MRL], gene content, and sequence alignments. The subgenomes assigned by intraspecific collinear blocks (dominant and recessive subgenomes of the eight Juglandaceae species and the five outgroups; dominant subgenome (D); recessive subgenome (R)). The Juglandaceae species are *Carya illinoinensis* (Cil), *Engelhardia roxburghiana* (Ero), *Juglans mandshurica* (Jma), *Juglans microcarpa* (Jmi), *Juglans regia* (Jre), *Pterocarya stenoptera* (Pste), *Platycarya strobilacea* (Pst), *Rhoiptelea chiliantha* (Rch) and the outgroups are *Betula pendula* (Bpe), *Carpinus fangiana* (Cfa), *Myrica rubra* (Mru), *Ostryopsis davidiana* (Oda), *Quercus lobata* (Qlo). The panel shows phylogenies inferred by

Syn-MRL under three different parameter settings, A_5G_{25} (A: the minimum number of anchor pairs required to call a collinear block, G: maximum number of intervening genes between two (adjacent) anchor pairs in collinear blocks) (**a**), $A_{10}G_{25}$ (**b**), and $A_{15}G_{25}$ (**c**), including both dominant and recessive subgenomes only dominant subgenomes, or only recessive subgenomes. The phylogenies inferred from gene presence/absence **(d)** or DNA-sequence-alignments (**e**) including both subgenomes, only dominant subgenomes, or only recessive subgenomes. Ultrafast bootstrap (UFBoot) support (%) is shown for each node in panels **(a–d)**, and local posterior probability is shown for each internal node in panel **(e)**.

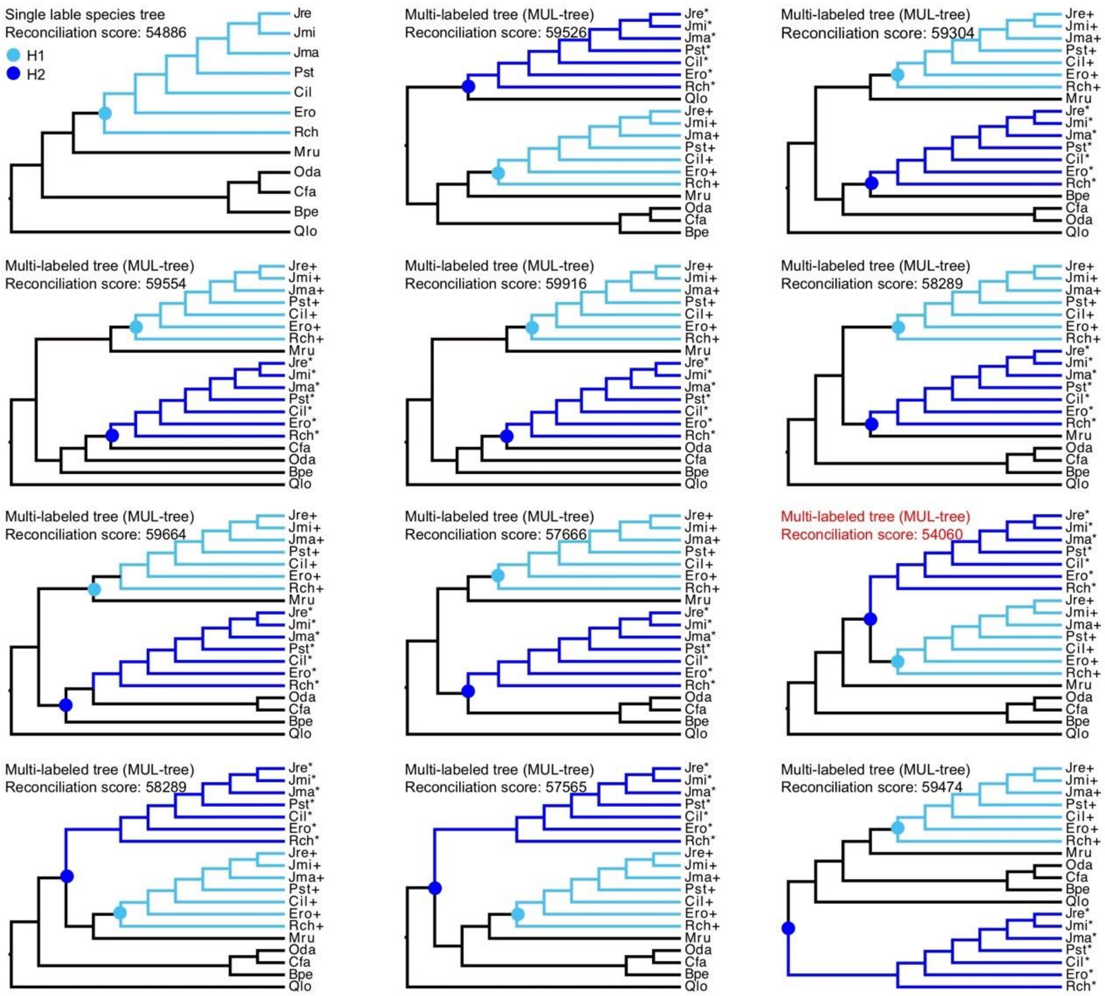

Supplementary Figure 15. The optimal multi-labelled tree (MUL-tree) inferred from GRAMPA analysis. Node H1 (cyan) defines the polyploid clade made up of seven species of Juglandaceae. Node H2 (blue) defines the location of the second parental lineage. We specified H1 node searching for H2 node in GRAMPA. The reconciliation scores are obtained by reconciliation of all gene trees (4,653 gene trees, bootstrap values ≥ 50) against both the species tree and all possible MUL trees. The Juglandaceae species are *Carya illinoinensis* (Cil), *Engelhardia roxburghiana* (Ero), *Juglans mandshurica* (Jma), *Juglans microcarpa* (Jmi), *Juglans regia* (Jre), *Platycarya strobilacea* (Pst), *Rhoiptelea chiliantha* (Rch) and the outgroups are *Betula pendula* (Bpe), *Carpinus fangiana* (Cfa), *Myrica rubra* (Mru), *Ostryopsis davidiana* (Oda), *Quercus lobata* (Qlo).

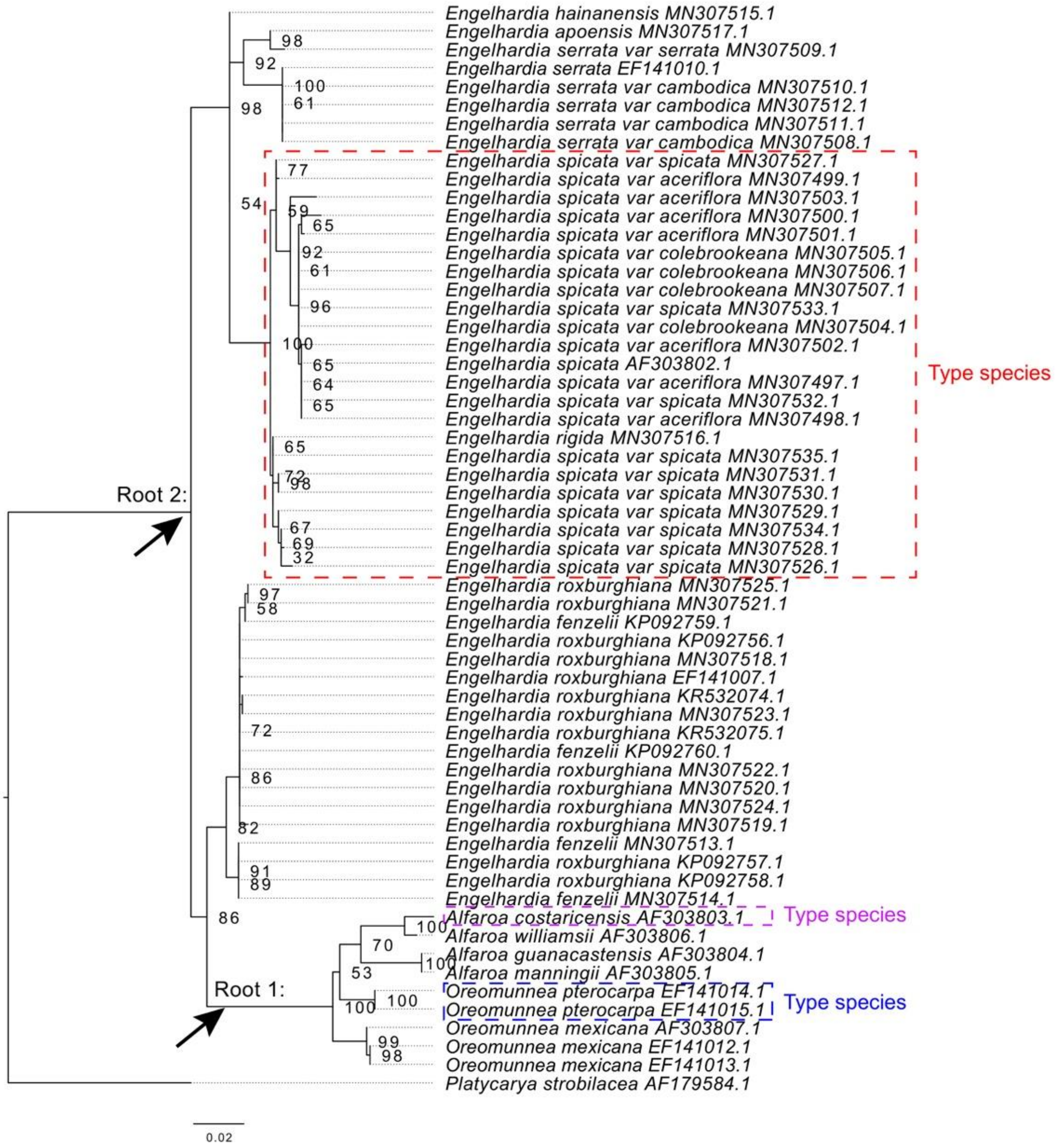

Supplementary Figure 16. A ML tree inferred from 59 herbarium-vouchered nuclear ITS sequences of Engelhardieae available in GenBank by May 2022. These sequences come from studies of Manos and Stone[38], Manos *et al*.[39], Liu *et al*.[40], and Zhang *et al*.[41]. ML bootstrap values (MLBS) >50% are labelled on each node.

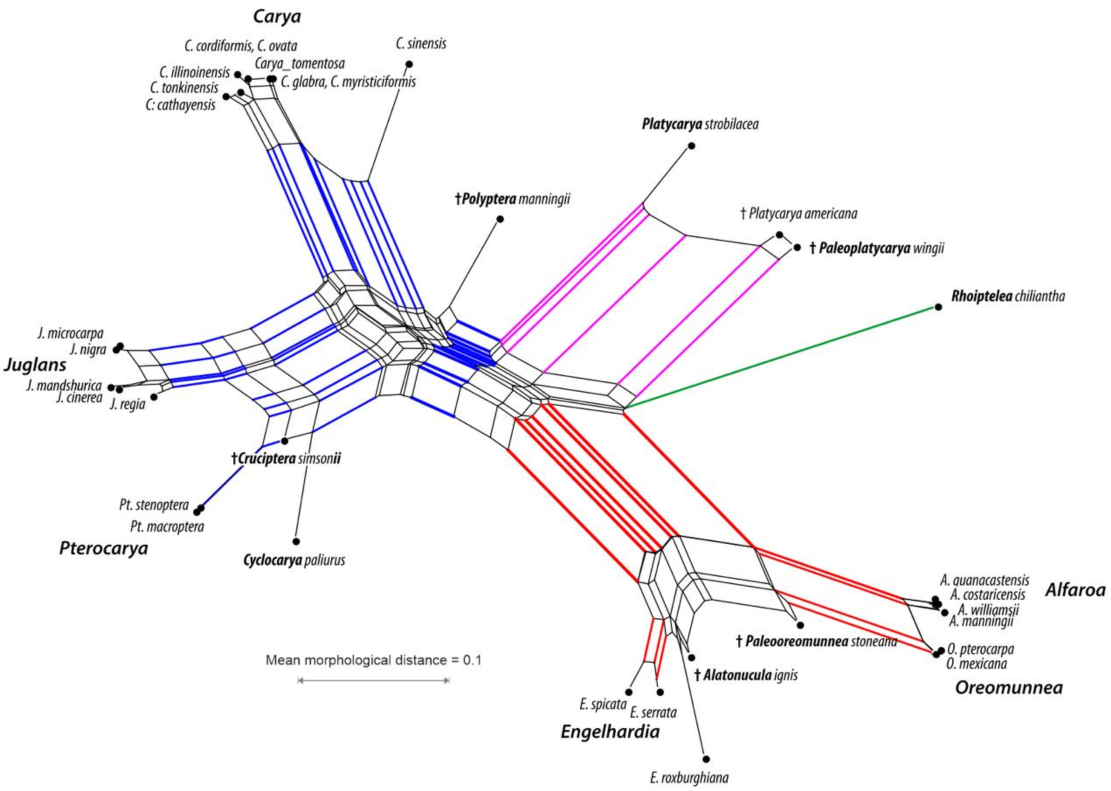

Supplementary Figure 17. A Neighbor-Net obtained from 64 morphological characters coded for 28 living and 6 extinct taxa by Hermsen and Gandolfo[42]. We also re-analysed the morphological matrices of Manos and Stone[38] of 64 characters coded for 40 living taxa, Larson-Johnson[43] of 89 characters for 37 living and 27 extinct taxa, and Zhang *et al*.[44] of 73 characters for 47 living species and 113 extinct taxa. All show *Platycarya* as an isolated lineage with ambiguous relationships. None of the four matrices include *Budvaricarpus serialis*, an 85 My-old fossil fruiting structure that represents *R. chiliantha*[45], and this might affect the position of the root.

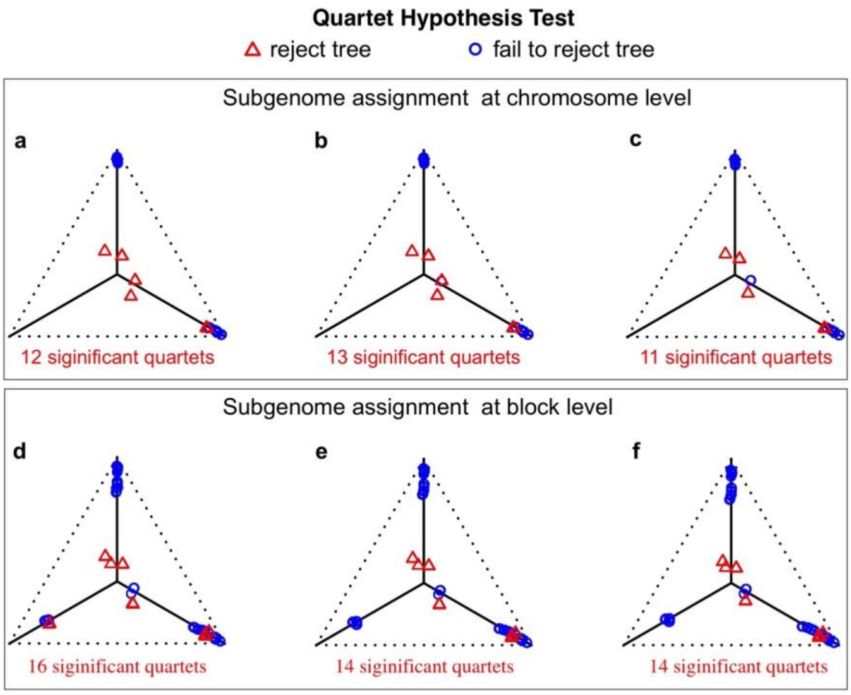

Supplementary Figure 18. Simplex plots of quartet concordance factors (qcCFs) under the multispecies coalescent (MSC) model of ILS and the T3 model (no specific species tree topology hypothesized) using the R package MSCquartets[46,47]. To minimize gene tree inference error, we perform MSCquartets analyses under different settings for minimum internal quartet branch length and after removing such loci that show a signal of recombination. If the branch length was less than 0, 10^{-5}, or 10^{-3} (in substitution unit), quartets were treated as polytomies. PhiPack v 1.1[48] was used to test for recombination for every gene in the datasets of orthologous genes determined under subgenome assignment at either chromosome level or block level, using permutation test with a *P*-value of <0.05 being treated as recombinant gene. We used the Holm-Bonferroni method to adjust for multiple testing with cutoff <0.05 for MSCquartets analyses. Red triangles in the plot represent rejection of the MSC model and indicate gene tree discord perhaps caused by introgression; blue circles represent a failure to reject the null hypothesis of MSC model. **(a)-(c)** represent the orthologous genes from the dominant and recessive subgenome of the seven Juglandaceae species whose subgenomes were assigned by homoeologous chromosomes (including *Carya illinoinensis*, *Engelhardia roxburghiana*, *Juglans mandshurica*, *Juglans microcarpa*, *Juglans regia*, *Platycarya strobilacea*, *Rhoiptelea chiliantha*). After removal of recombinant loci, there were 5,882 gene trees for seven taxa and 35 different four-taxon sets in the MSCquartets analysis. **(d)-(f)** represent the orthologous genes from the dominant and recessive subgenomes of the eight Juglandaceae species whose subgenomes were assigned by intraspecific collinear blocks (allowing us to include one additional species, *Pterocarya stenoptera*, which was assembled at the scaffold level). There were 4,679 gene trees after removing recombinant loci and 70 different four-taxon sets in the MSCquartets analysis.

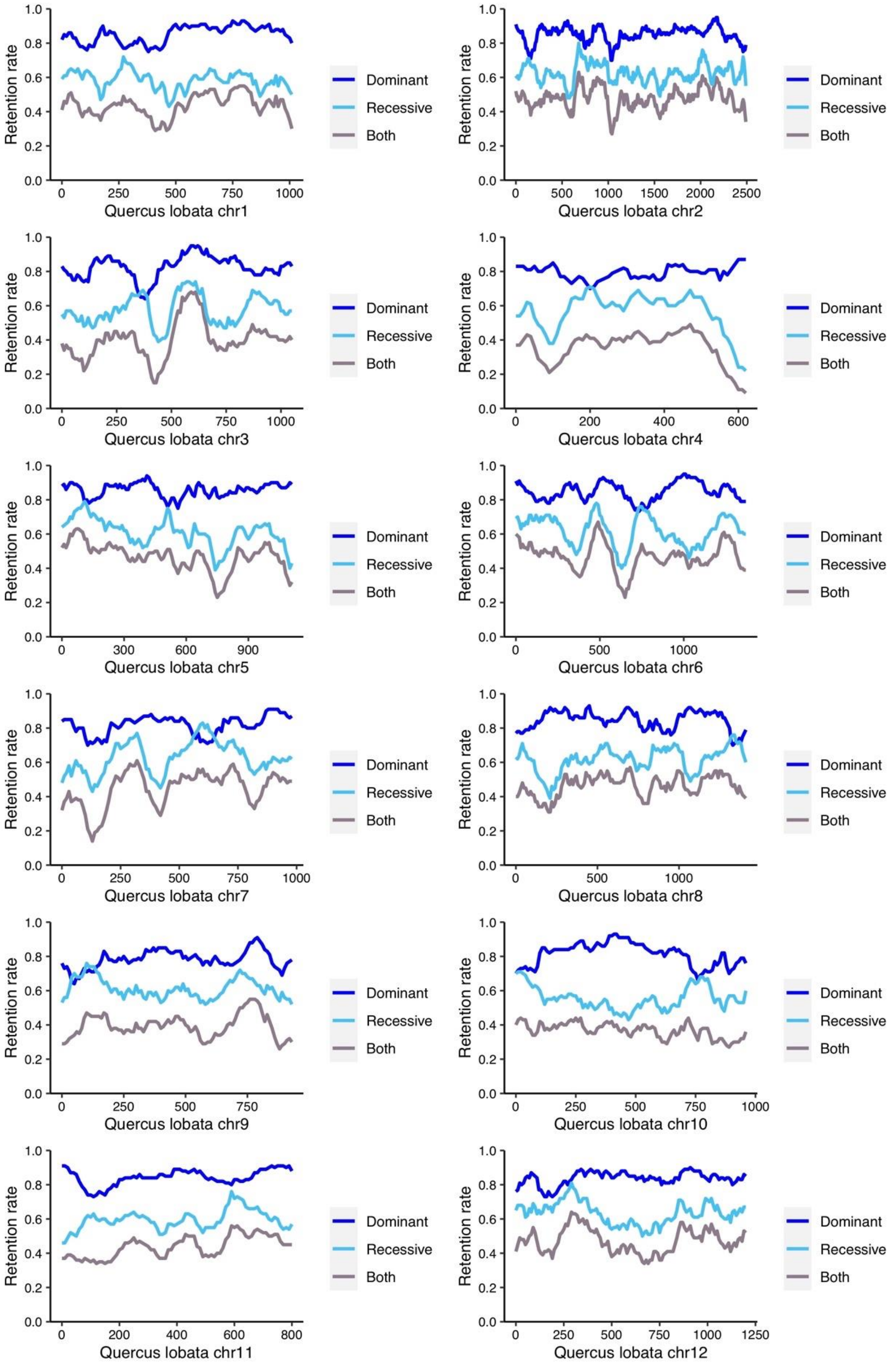

Supplementary Figure 19. Fractionation pattern on the dominant and recessive subgenome of *Carya illinoinensis* using *Quercus lobata* as the target genome. The X axis indicates gene locations along each *Q. lobata* chromosome, and the Y axis indicates the proportion of orthologous syntenic genes retained (retention rate) in *C. illinoinensis* dominant subgenome (blue), recessive subgenome (cyan) and both subgenomes (gray), corresponding to *Q. lobata* chromosomes. The percentage of retained orthologous genes in *C. illinoinensis* was calculated based on 100-gene sliding windows along each *Q. lobata* chromosome.

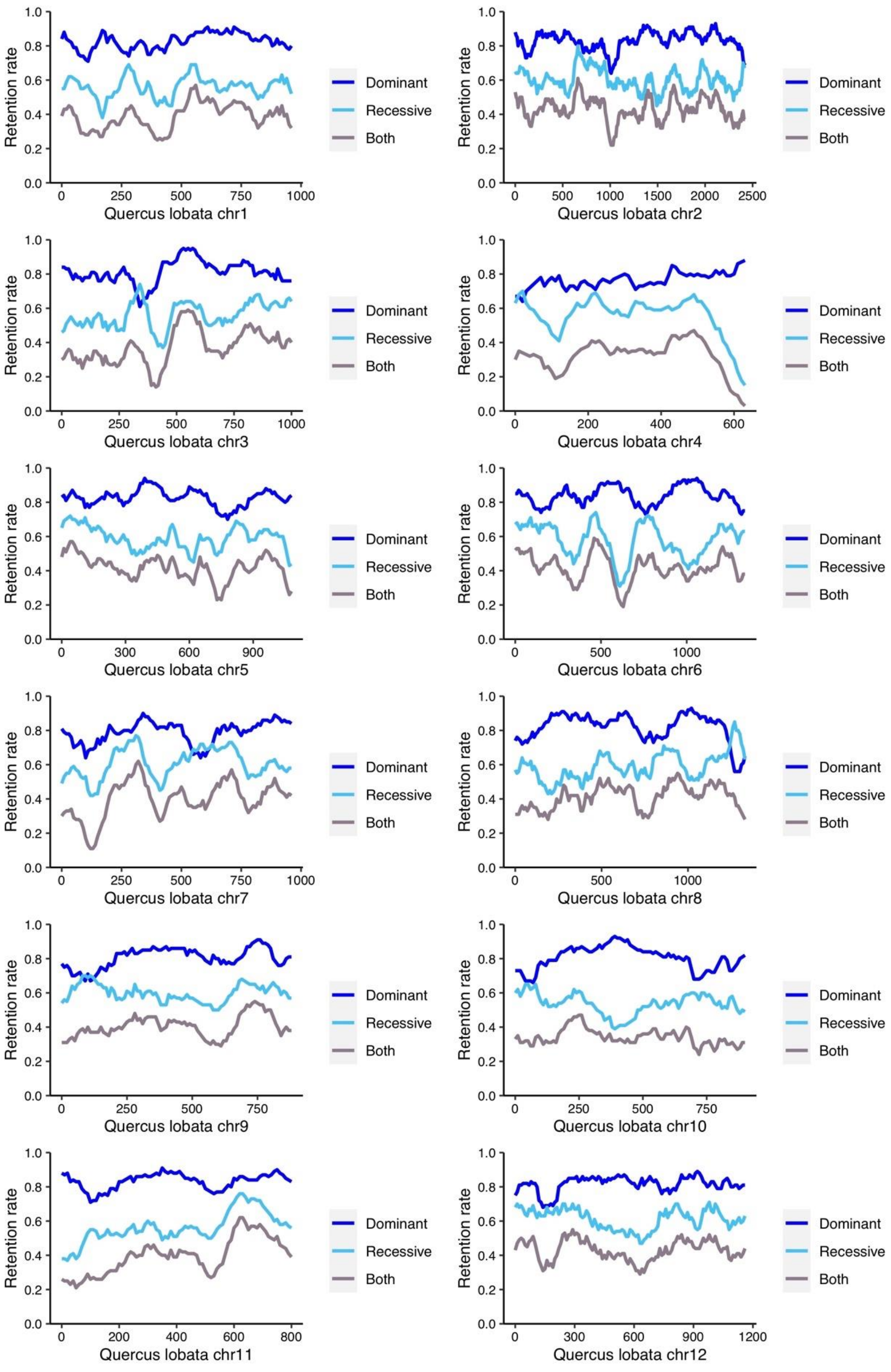

Supplementary Figure 20. Fractionation pattern on the dominant and recessive subgenome of *Engelhardia roxburghiana* using *Quercus lobata* as the target genome. The X axis indicates gene locations along each *Q. lobata* chromosome, and the Y axis indicates the proportion of orthologous syntenic genes retained (retention rate) in *E. roxburghiana* dominant subgenome (blue), recessive subgenome (cyan) and both subgenomes (gray), corresponding to *Q. lobata* chromosomes. The percentage of retained orthologous genes in *E. roxburghiana* was calculated based on 100-gene sliding windows along each *Q. lobata* chromosome.

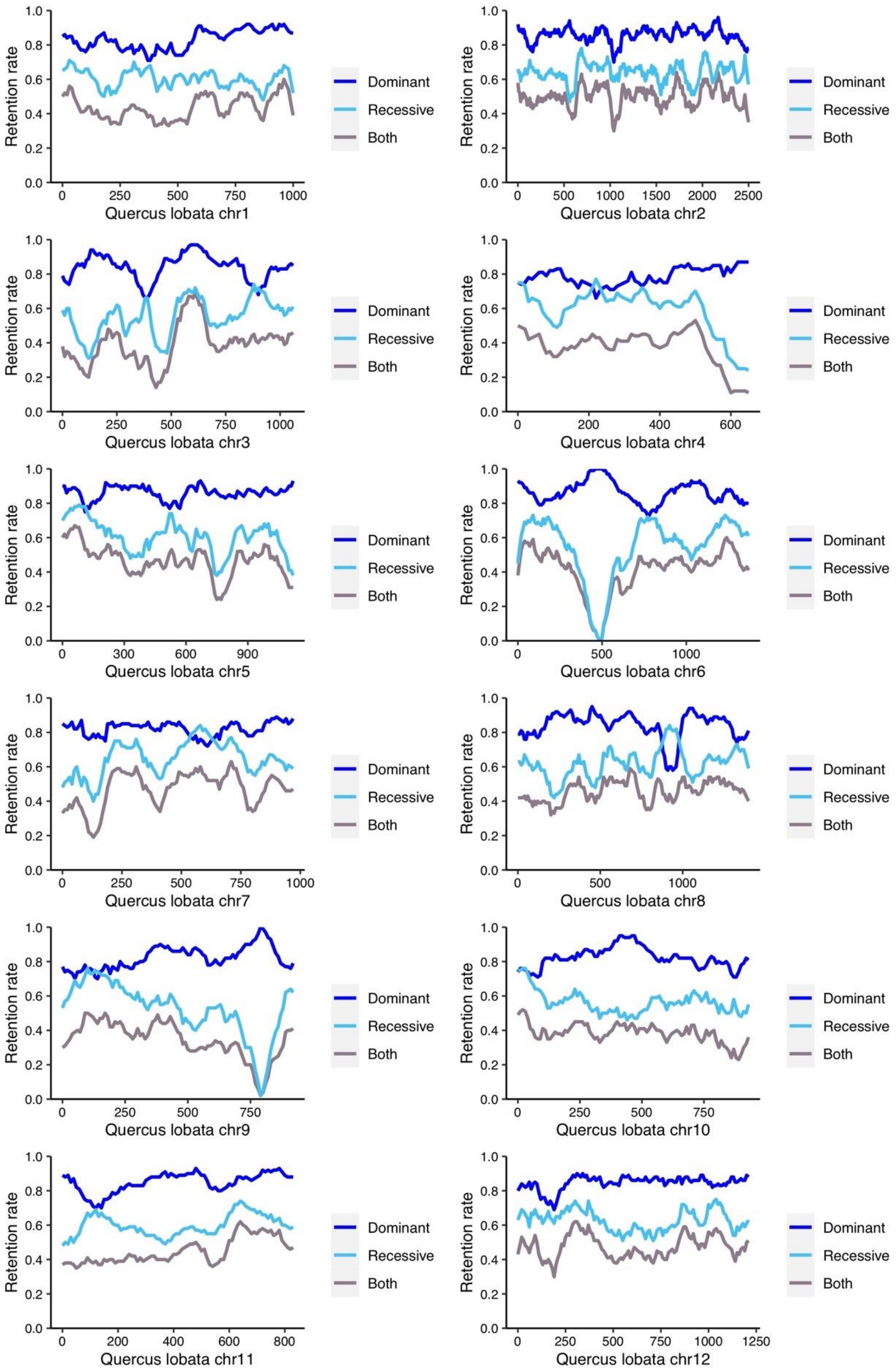

Supplementary Figure 21. Fractionation pattern on the dominant and recessive subgenome of *Juglans mandshurica* using *Quercus lobata* as the target genome. The X axis indicates gene locations along each *Q. lobata* chromosome, and the Y axis indicates the proportion of orthologous syntenic genes retained (retention rate) in *J. mandshurica* dominant subgenome (blue), recessive subgenome (cyan) and both subgenomes (gray), corresponding to *Q. lobata* chromosomes. The percentage of retained orthologous genes in *J. mandshurica* was calculated based on 100-gene sliding windows along each *Q. lobata* chromosome.

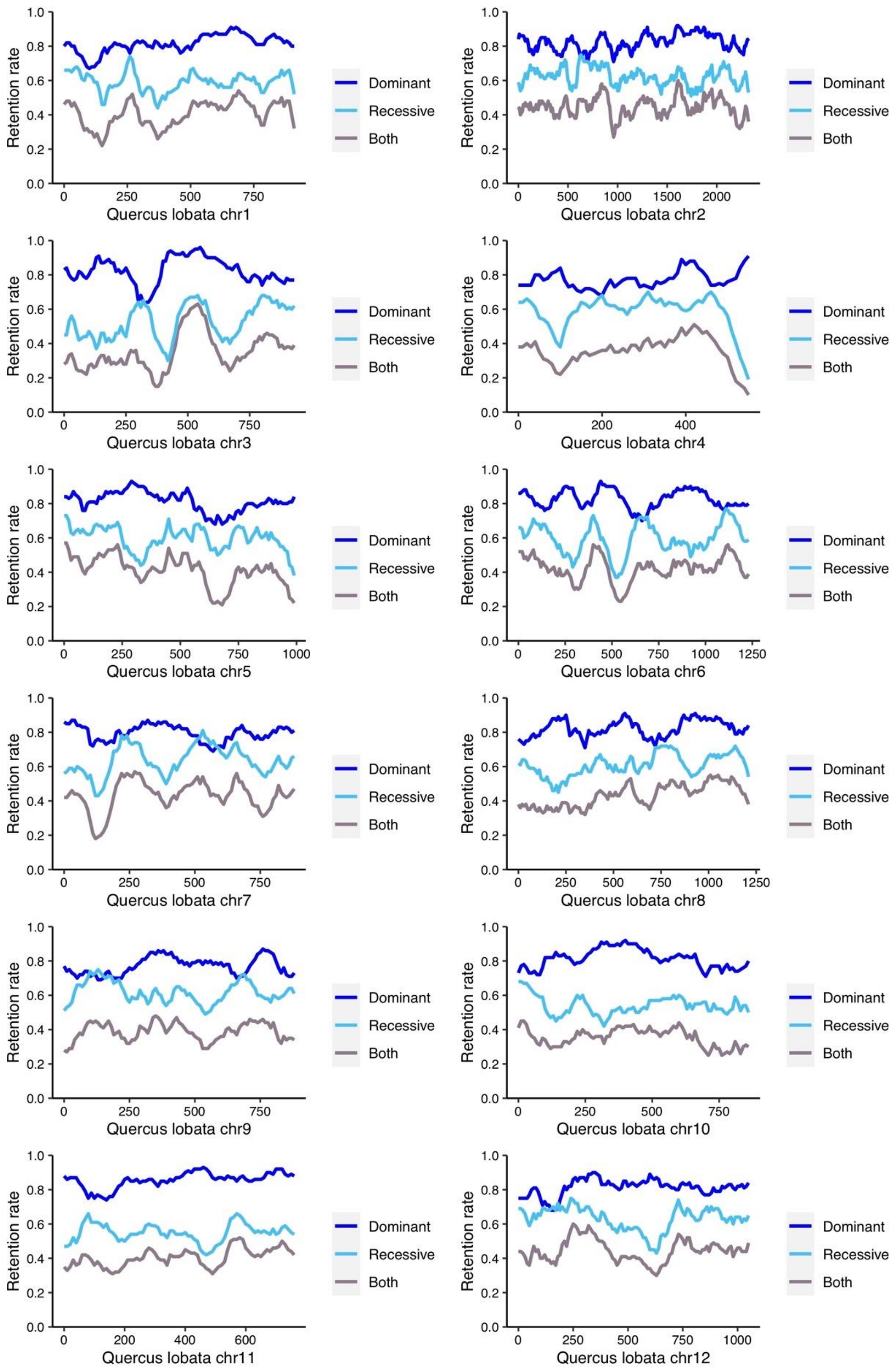

Supplementary Figure 22. Fractionation pattern on the dominant and recessive subgenome of *Juglans microcarpa* using *Quercus lobata* as the target genome. The X axis indicates gene locations along each *Q. lobata* chromosome, and the Y axis indicates the proportion of orthologous syntenic genes retained (retention rate) in *J. microcarpa* dominant subgenome (blue), recessive subgenome (cyan) and both subgenomes (gray), corresponding to *Q. lobata* chromosomes. The percentage of retained orthologous genes in *J. microcarpa* was calculated based on 100-gene sliding windows along each *Q. lobata* chromosome.

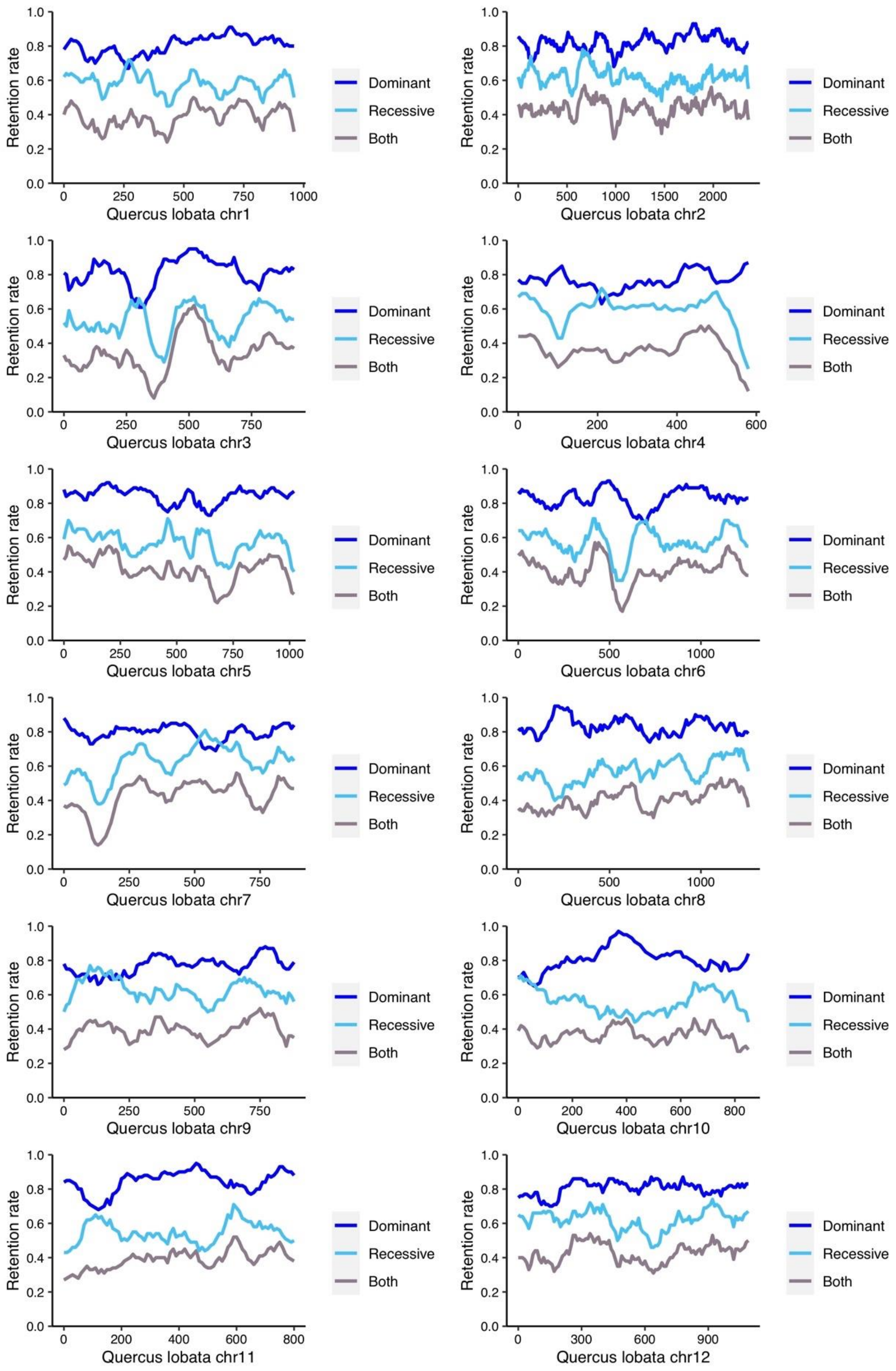

Supplementary Figure 23. Fractionation pattern on the dominant and recessive subgenome of *Juglans regia* using *Quercus lobata* as the target genome. The X axis indicates gene locations along each *Q. lobata* chromosome, and the Y axis indicates the proportion of orthologous syntenic genes retained (retention rate) in *J. regia* dominant subgenome (blue), recessive subgenome (cyan) and both subgenomes (gray), corresponding to *Q. lobata* chromosomes. The percentage of retained orthologous genes in *J. regia* was calculated based on 100-gene sliding windows along each *Q. lobata* chromosome.

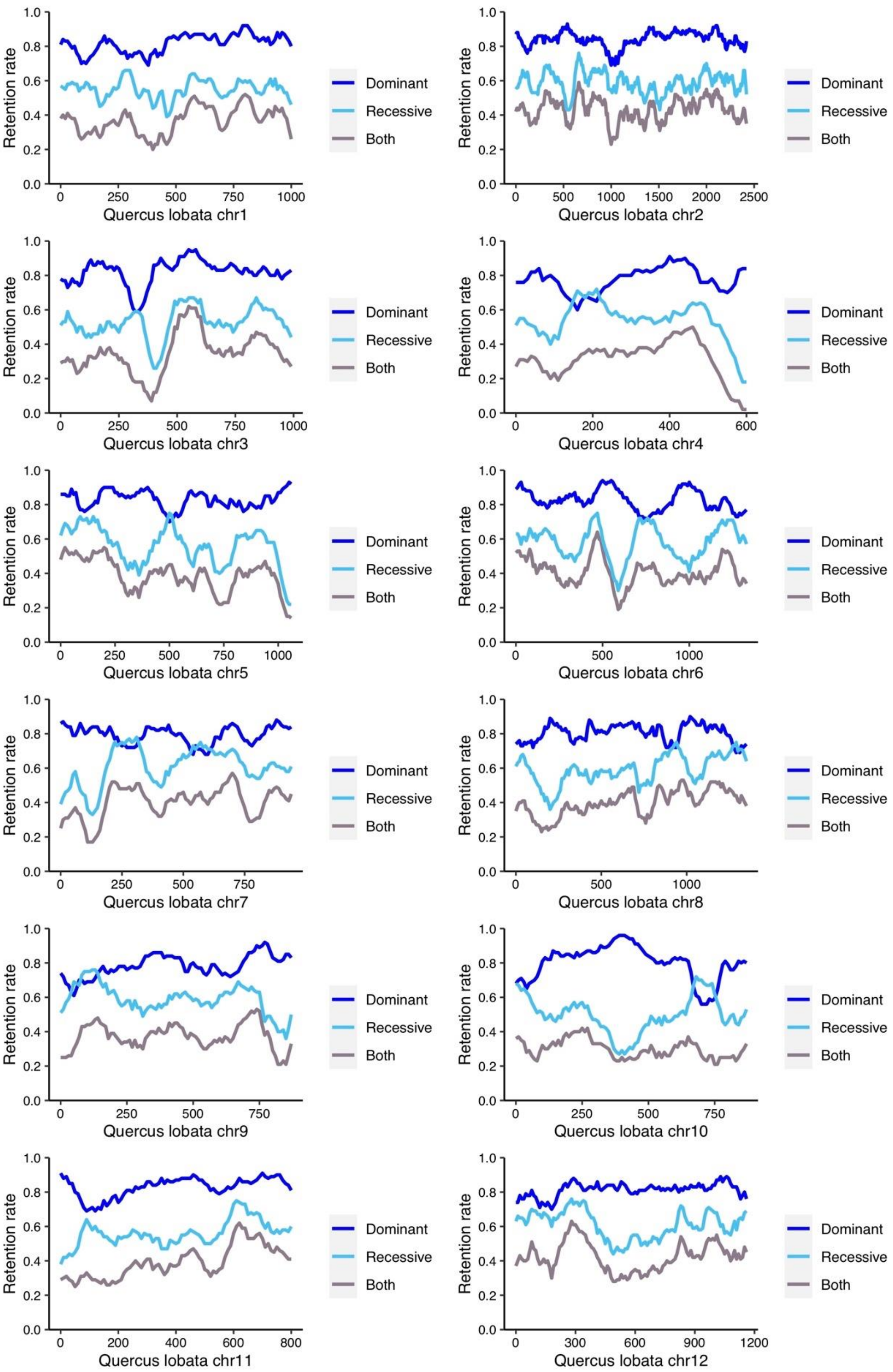

Supplementary Figure 24. Fractionation pattern on the dominant and recessive subgenome of *Platycarya strobilacea* using *Quercus lobata* as the target genome. The X axis indicates gene locations along each *Q. lobata* chromosome, and the Y axis indicates the proportion of orthologous syntenic genes retained (retention rate) in *P. strobilacea* dominant subgenome (blue), recessive subgenome (cyan) and both subgenomes (gray), corresponding to *Q. lobata* chromosomes. The percentage of retained orthologous genes in *P. strobilacea* was calculated based on 100-gene sliding windows along each *Q. lobata* chromosome.

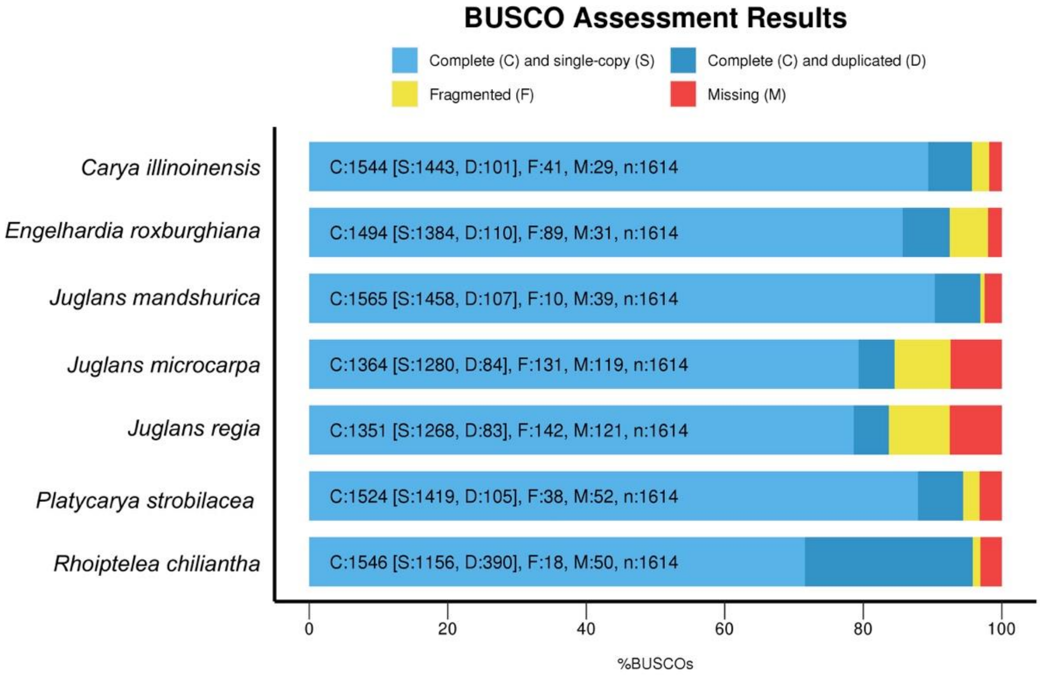

Supplementary Figure 25. Assessment of completeness of gene annotations for the seven Juglandaceae species, *C. illinoinensis, E. roxburghiana, J. mandshurica, J. microcarpa, J. regia, P. strobilacea* and *R. chiliantha*.

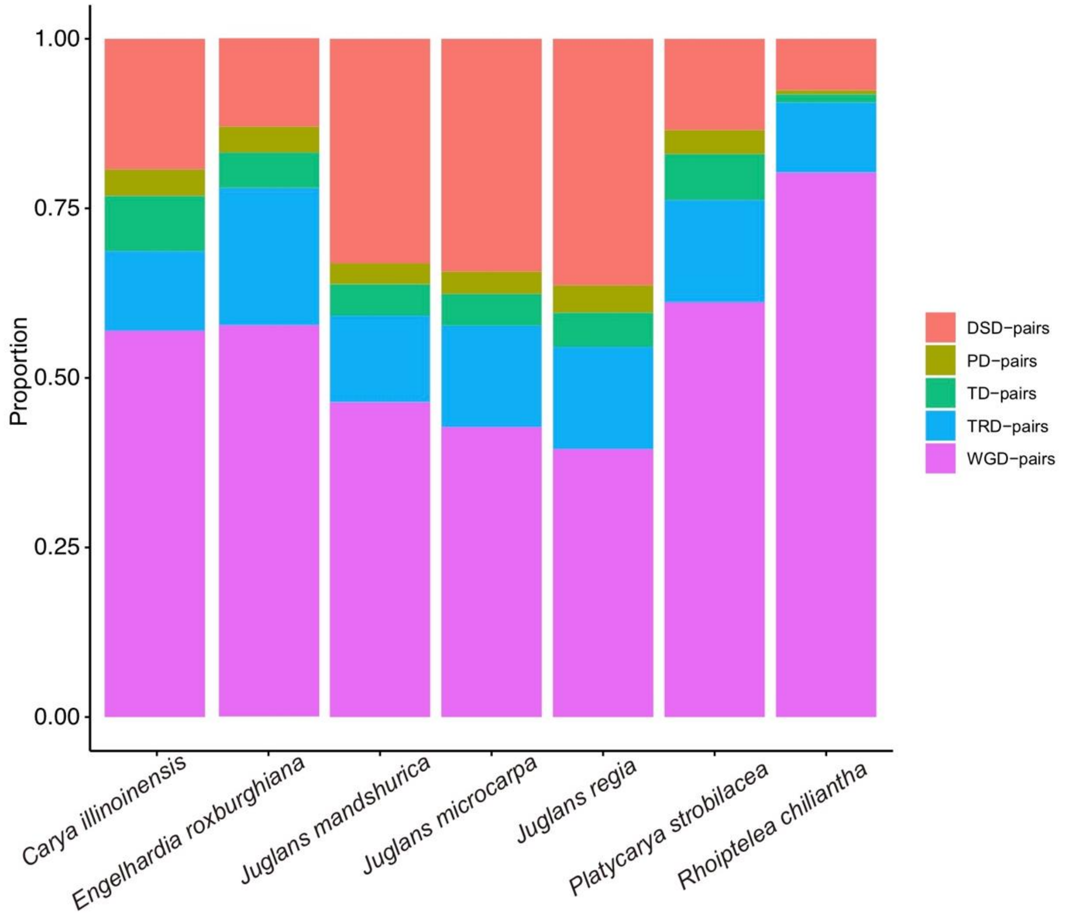

Supplementary Figure 26. The proportion of gene pairs derived from different modes of duplication. WGD-pairs: whole-genome duplication gene pairs; TD-pairs: tandem gene pairs; PD-pairs: proximal gene pairs; TRD-pairs: transposed gene pairs; DSD-pairs: dispersed gene pairs.

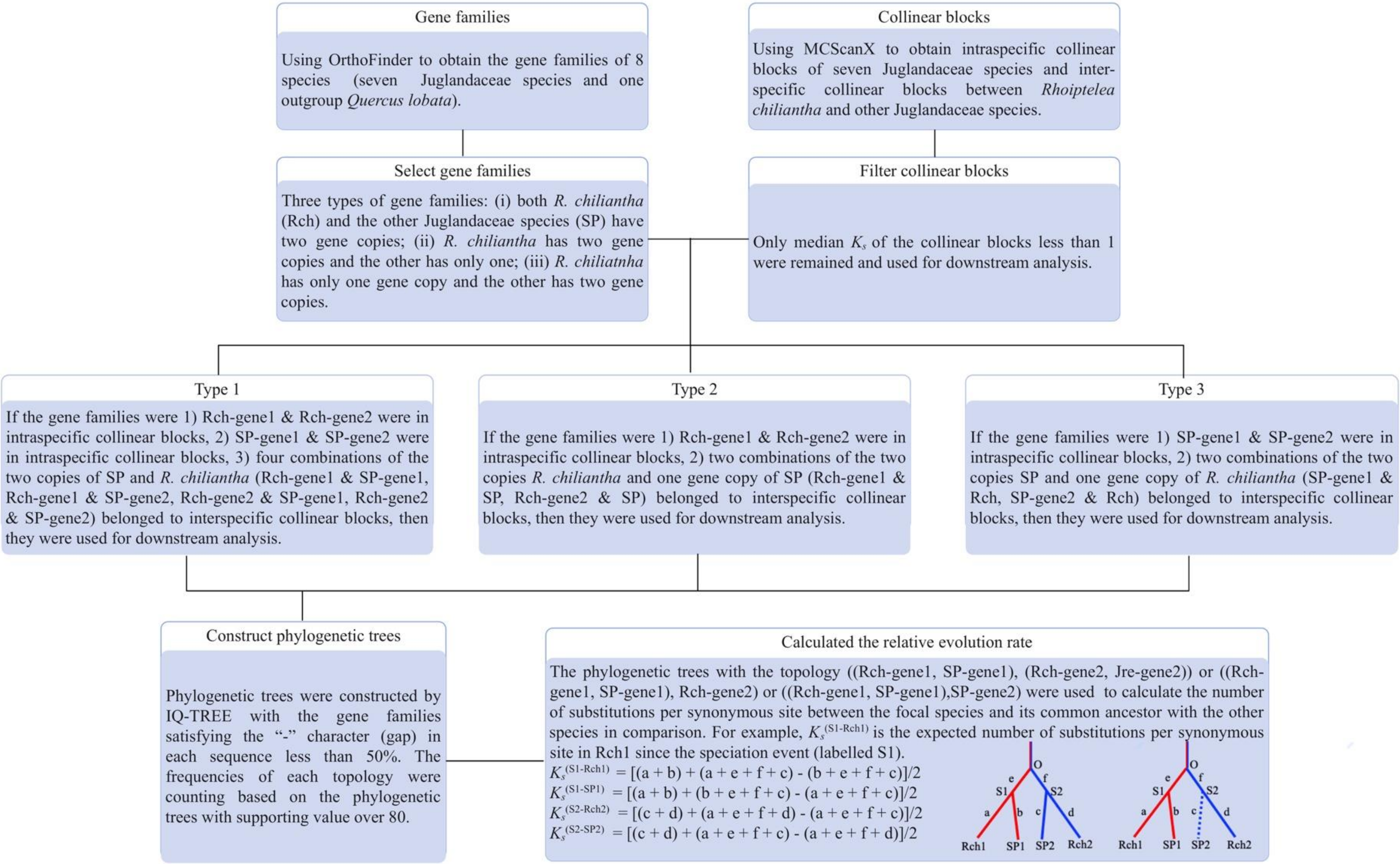

Supplementary Figure 27. Workflow for choosing gene families to detect whether *R. chiliantha* shared WGD with other species of Juglandaceae and for calculating the relative evolutionary rate.

Supplementary Table 1. Genome information for assembled and downloaded genomes.

Species	Assembly size (Mbp)	Scaffold N50 (Mbp)	Number of annotated genes	Repeat sequence (%)	URLs	SRA accession number
Rhoiptelea chiliantha	408.19	24.34	32,505	39.35	http://cmb.bnu.edu.cn/juglans/	SRR18156254
Engelhardia roxburghiana	884.78	54.38	30,590	57.11	http://cmb.bnu.edu.cn/juglans/	SRR18156253
Platycarya strobilacea	695.01	45.53	29,330	46.09	http://cmb.bnu.edu.cn/juglans/	SRR9313657
Juglans regia	534.67	35.20	31,425	44.15	http://aegilops.wheat.ucdavis.edu/Walnut/annotation/	SRR6382584
Juglans microcarpa	527.90	35.63	29,496	43.88	http://aegilops.wheat.ucdavis.edu/Walnut/annotation/	SRR5097499
Juglans mandshurica	537.15	35.99	34,621	50.10	http://cmb.bnu.edu.cn/juglans/	SRR15113168
Carya illinoinensis	674.27	44.71	32,267	49.70	https://phytozomenext.jgi.doe.gov/info/CillinoinensisPawnee_v1_1	SRR13094339

Supplementary Table 2. Genome completeness measured by Benchmarking Universal Single-Copy Orthologs (BUSCO).

	R. chiliantha		*E. roxburghiana*	
	**Number**	**Ratio**	**Number**	**Ratio**
Complete BUSCOs (C)	1,384	96.1%	1,349	93.7%
Complete and single-copy BUSCOs (S)	964	66.9%	1,207	83.8%
Complete and duplicated BUSCOs (D)	420	29.2%	142	9.9%
Fragmented BUSCOs (F)	14	1.0%	25	1.7%
Missing BUSCOs (M)	42	2.9%	66	4.6%
Total	1,440		1,440	

Supplementary Table 3. The peaks of K_s estimated by the relative test and molecular evolution rate (μ) of the seven Juglandaceae species in this study after the juglandoid WGD.

Species	Peak of K_s	μ (× 10^{-9}) (per site per year)
R. chiliantha	0.047	0.553
E. roxburghiana	0.166	1.952
C. illinoinensis	0.136	1.600
P. strobilacea	0.161	1.894
J. regia	0.128	1.506
J. microcarpa	0.131	1.541
J. mandshurica	0.125	1.471

Supplementary Table 4. The heterozygosity and effective population size of the seven Juglandaceae species in this study.

**Sample**	**Species**	**Depth (×)**	**Heterozygosity**	N_e
Rch-ref	*R. chiliantha*	62	0.60×10^{-3}	8,919
Aro-ref	*E. roxburghiana*	29	5.16×10^{-3}	22,036
Cil-SRR13094339	*C. illinoinensis*	43	4.94×10^{-3}	25,737
Plst-str	*P. strobilacea*	30	4.09×10^{-3}	17,974
Jre-R1	*J. regia*	45	1.93×10^{-3}	10,701
Jmi-386.01A	*J. microcarpa*	34	3.89×10^{-3}	21,007
Jma-AT12	*J. mandshurica*	30	3.76×10^{-3}	21,326

Supplementary Table 5. The analysis of RNA-seq data for six DNA repair and recombination genes in the three tissues of *J. regia*.

Gene name	Catkin (FPKM[a])	Pistillate flower (FPKM[a])	Leaf (FPKM[a])			
	SRR1068161	SRR1068421	SRR1067933	SRR1068165	SRR1068166	SRR1068437
Jr5DG00013500	2.84	5.27	4.41	2.50	3.42	4.06
Jr7DG00038800	7.93	13.96	14.12	5.63	12.55	9.89
Jr4DG00208600	36.00	16.41	87.10	47.55	80.52	82.83
Jr2SG00141900	63.55	105.37	54.78	46.39	49.87	43.28
Jr2DG00194400	1.56	51.53	10.87	0.22	11.00	11.52
Jr2DG00192200	0.44	5.89	1.91	0.23	1.73	1.56

[a]FPKM: Fragments Per Kilobase of exon model per Million mapped fragments.

Supplementary Table 6. Details for the three approaches to infer parental lineages and subgenome relationships in the Juglandaceae, viz. microsynteny, gene content, and DNA alignment.

Subgenome assignment	Subgenome	Microsynteny					Gene content	Alignment
		Parameter settings	Number of collinear blocks	Number of nodes	Number of edges	Size of matrix (rows×columns)	Size of matrix (rows×columns)	Number of gene family trees
Homoeologous chromosomes	Dominant+Recessive	[a]A_5G_{25}	67,954	270,276	1,976,821	19 × 19,202	19 × 17,149	3,927
	Dominant		36,460	192,958	995,563	12 × 17,926	12 × 17,926	8,639
	Recessive		29,224	163,052	106,324	12 × 16,894	12 × 16,894	6,127
	Dominant+Recessive	$A_{10}G_{25}$	31205	259,432	1,691,909	19 × 19,559		
	Dominant		20255	183888	846957	12 × 18,076		
	Recessive		12576	153831	597447	12 × 17,724		
	Dominant+Recessive	$A_{15}G_{25}$	20127	253842	1543617	19 × 20,255		
	Dominant		10928	178750	764191	12 × 18,764		
	Recessive		8002	148812	536695	12 × 18,379		
Intraspecific collinear blocks	Dominant+Recessive	A_5G_{25}	76,274	283,090	2,291,122	21 × 18,910	21 × 16895	3,956
	Dominant		39548	199,155	1,112,931	13 × 17,316	13 × 17701	6150
	Recessive		32056	169,575	814,258	13 × 16,921	13 × 16660	5573
	Dominant+Recessive	$A_{10}G_{25}$	35,581	272,881	1,974,429	21 × 19,323		
	Dominant		18,850	190,509	953,205	13 × 17,830		
	Recessive		13,990	160,420	675,631	13 × 17,645		
	Dominant+Recessive	$A_{15}G_{25}$	23,352	267,441	1,810,328	21 × 19,988		
	Dominant		12,226	185,537	864,984	13 × 18,487		
	Recessive		9,035	155,368	609,769	13 × 18,269		

[a]A: the minimum number of anchor pairs required to call a collinear block, G: maximum number of intervening genes between two (adjacent) anchor pairs in collinear blocks.

Supplementary Table 7. The different modes of gene duplication in seven Juglandaceae species.

Species	WGD-pairs	TD-pairs	PD-pairs	TRD-pairs	DSD-pairs
Rhoiptelea chiliantha	17,733	278	128	2,277	1,672
Engelhardia roxburghiana	9,712	886	643	3,406	2,191
Platycarya strobilacea	9,567	1,064	556	2,363	2,102
Carya illinoinensis	11,220	1,608	772	2,318	3,791
Juglans regia	8,870	1,142	901	3,389	8,160
Juglans microcarpa	8,966	986	692	3,135	7,194
Juglans mandshurica	11,260	1,127	745	3,098	8,028

WGD-pairs: whole-genome duplication gene pairs; TD-pairs: tandem gene pairs; PD-pairs: proximal gene pairs; TRD-pairs: transposed gene pairs; DSD-pairs: dispersed gene pairs.

Supplementary references

1. Schnable, J. C., Springer, N. M. & Freeling, M. Differentiation of the maize subgenomes by genome dominance and both ancient and ongoing gene loss. *Proc. Natl. Acad. Sci. USA* **108**, 4069–4074 (2011).
2. Edger, P. P., McKain, M. R., Bird, K. A. & VanBuren, R. Subgenome assignment in allopolyploids: Challenges and future directions. *Curr. Opin. Plant Biol.* **42**, 76–80 (2018).
3. Shi, T. et al. Distinct expression and methylation patterns for genes with different fates following a single whole-genome duplication in flowering plants. *Molecular Biology Evolution* **37**, 2394–2413 (2020).
4. Sork, V. L. et al. High-quality genome and methylomes illustrate features underlying evolutionary success of oaks. *Nat. Commun.* **13**, 2047 (2022).
5. Lovell, J. T. et al. Four chromosome scale genomes and a pan-genome annotation to accelerate pecan tree breeding. *Nat. Commun.* **12**, 1–12 (2021).
6. Zhu, T. et al. Sequencing a *Juglans regia* x *J. microcarpa* hybrid yields high-quality genome assemblies of parental species. *Hortic. Res.* **6**, 55 (2019).
7. Zhang, W. P. et al. Dead-end hybridization in walnut trees revealed by large-scale genomic sequence data. *Mol. Biol. Evol.* **39**, msab308 (2022).
8. Zhang, B. W. et al. Phylogenomics reveals an ancient hybrid origin of the Persian walnut. *Mol. Biol. Evol.* **36**, 2451–2461 (2019).
9. Chin, C. S. et al. Phased diploid genome assembly with single-molecule real-time sequencing. *Nat. Methods* **13**, 1050–1054 (2016).
10. Li, H. & Durbin, R. Fast and accurate short read alignment with Burrows-Wheeler transform. *Bioinformatics* **25**, 1754–1760 (2009).
11. Walker, B. J. et al. Pilon: an integrated tool for comprehensive microbial variant detection and genome assembly improvement. *PLoS One* **9**, e112963 (2014).
12. Wingett, S. et al. HiCUP: Pipeline for mapping and processing Hi-C data. *F1000Research* **4**, 1310 (2015).
13. Zhang, X., Zhang, S., Zhao, Q., Ming, R. & Tang, H. Assembly of allele-aware, chromosomal-scale autopolyploid genomes based on Hi-C data. *Nat. Plants.* **5**, 833–845 (2019).
14. Robinson, J. T. et al. Juicebox. js provides a cloud-based visualization system for Hi-C data. *Cell Syst.* **6**, 256–258. e251 (2018).
15. Simão, F. A., Waterhouse, R. M., Ioannidis, P., Kriventseva, E. V. & Zdobnov, E. M. BUSCO: Assessing genome assembly and annotation completeness with single-copy orthologs. *Bioinformatics* **31**, 3210–3212 (2015).
16. Benson, G. Tandem repeats finder: A program to analyze DNA sequences. *Nucleic Acids Res.* **27**, 573–580 (1999).

17. Jurka, J. Repbase update: A database and an electronic journal of repetitive elements. *Trends Genet.* **16**, 418–420 (2000).
18. Chen, N. Using RepeatMasker to Identify repetitive elements in genomic sequences. *Curr. Protoc. Bioinf.* **5**, 4.10. 11–14.10. 14 (2004).
19. Xu, Z. & Wang, H. LTR_FINDER: An efficient tool for the prediction of full-length LTR retrotransposons. *Nucleic Acids Res.* **35**, W265–W268 (2007).
20. Price, A. L., Jones, N. C. & Pevzner, P. A. De novo identification of repeat families in large genomes. *Bioinformatics* **21**, i351–i358 (2005).
21. Edgar, R. C. Search and clustering orders of magnitude faster than BLAST. *Bioinformatics* **26**, 2460–2461 (2010).
22. Salojärvi, J. et al. Genome sequencing and population genomic analyses provide insights into the adaptive landscape of silver birch. *Nat. Genet.* **49**, 904–912 (2017).
23. Huang, Y. J. et al. The genomes of pecan and Chinese hickory provide insights into *Carya* evolution and nut nutrition. *GigaScience* **8**, giz036 (2019).
24. Jia, H. M. et al. The red bayberry genome and genetic basis of sex determination. *Plant Biotechnol. J.* **17**, 397–409 (2019).
25. Yang, Y. Z. et al. Genomic effects of population collapse in a critically endangered ironwood tree *Ostrya rehderiana*. *Nat. Commun.* **9**, 1–9 (2018).
26. Birney, E., Clamp, M. & Durbin, R. GeneWise and Genomewise. *Genome Res.* **14**, 988–995 (2004).
27. Blanco, E., Parra, G. & Guigó, R. Using geneid to identify genes. *Curr. Protoc. Bioinf.* **18**, 4.3.1–4.3.28 (2007).
28. Ramakrishna, R. & Srinivasan, R. Gene identification in bacterial and organellar genomes using GeneScan. *Comput. Biol. Chem.* **23**, 165–174 (1999).
29. Delcher, A. L., Harmon, D., Kasif, S., White, O. & Salzberg, S. L. Improved microbial gene identification with GLIMMER. *Nucleic Acids Res.* **27**, 4636–4641 (1999).
30. Korf, I. Gene finding in novel genomes. *BMC Bioinf.* **5**, 1–9 (2004).
31. Kim, D., Langmead, B. & Salzberg, S. L. HISAT: A fast spliced aligner with low memory requirements. *Nat. Methods* **12**, 357–360 (2015).
32. Pertea, M. et al. StringTie enables improved reconstruction of a transcriptome from RNA-seq reads. *Nat. Biotechnol.* **33**, 290–295 (2015).
33. Haas, B. J. et al. Automated eukaryotic gene structure annotation using EVidenceModeler and the Program to Assemble Spliced Alignments. *Genome Biol.* **9**, 1–22 (2008).
34. Emms, D. M. & Kelly, S. OrthoFinder: phylogenetic orthology inference for comparative genomics. *Genome Biol.* **20**, 1–14 (2019).

35. Wang, Y. et al. MCScanX: a toolkit for detection and evolutionary analysis of gene synteny and collinearity. *Nucleic Acids Res.* **40**, e49 (2012).
36. Wang, D., Zhang, Y., Zhang, Z., Zhu, J. & Yu, J. KaKs_Calculator 2.0: A toolkit incorporating gamma-series methods and sliding window strategies. *Genomics Proteomics Bioinformatics* **8**, 77–80 (2010).
37. Minh, B. Q. et al. IQ-TREE 2: New models and efficient methods for phylogenetic inference in the genomic era. *Mol. Biol. Evol.* **37**, 1530–1534 (2020).
38. Manos, P. S. & Stone, D. E. Evolution, phylogeny, and systematics of the Juglandaceae. *Ann. Mo. Bot. Gard.* **88**, 231–269 (2001).
39. Manos, P. S. et al. Phylogeny of extant and fossil Juglandaceae inferred from the integration of molecular and morphological data sets. *Syst. Biol.* **56**, 412–430 (2007).
40. Liu, J. et al. The use of DNA barcoding as a tool for the conservation biogeography of subtropical forests in China. *Divers. Distrib.* **21**, 188–199 (2015).
41. Zhang, C.-Y. et al. Shining a light on species delimitation in the tree genus *Engelhardia* Leschenault ex Blume (Juglandaceae). *Mol. Phylogenet. Evol.* **152**, 106918 (2020).
42. Hermsen, E. J. & Gandolfo, M. A. Fruits of Juglandaceae from the Eocene of South America. *Syst. Bot.* **41**, 316–328 (2016).
43. Larson-Johnson, K. Phylogenetic investigation of the complex evolutionary history of dispersal mode and diversification rates across living and fossil Fagales. *New Phytol.* **209**, 418–435 (2016).
44. Zhang, Q., Ree, R. H., Salamin, N., Xing, Y. & Silvestro, D. Fossil-informed models reveal a Boreotropical origin and divergent evolutionary trajectories in the walnut family (Juglandaceae). *Syst. Biol.* **1**, 242–258 (2022).
45. Heřmanová, Z., Kvaček, J. & Friis, E. M. *Budvaricarpus serialis* Knobloch & Mai, an unusual new member of the Normapolles complex from the Late Cretaceous of the Czech Republic. *Int. J. Plant Sci.* **172**, 285–293 (2011).
46. Allman, E. S., Mitchell, J. D. & Rhodes, J. A. Gene tree discord, simplex plots, and statistical tests under the coalescent. *Syst. Biol.* **71**, 929–942 (2022).
47. Rhodes, J. A., Banos, H., Mitchell, J. D. & Allman, E. S. MSCquartets 1.0: quartet methods for species trees and networks under the multispecies coalescent model in R. *Bioinformatics* **37**, 1766–1768 (2021).
48. Bruen, T. C., Philippe, H. & Bryant, D. A simple and robust statistical test for detecting the presence of recombination. *Genetics* **172**, 2665–2681 (2006).
