# Supplementary material for: Genome structure-based Juglandaceae phylogenies contradict alignment-based phylogenies and substitution rates vary with DNA repair genes
Source: Nat Commun. 2023 Feb 4;14:617. doi: 10.1038/s41467-023-36247-z (PMC9899254; doi:10.1038/s41467-023-36247-z)
Supplement: Supplementary file 3 — Description of Additional Supplementary Files [file 41467_2023_36247_MOESM3_ESM.pdf]

Description of Additional Supplementary Files

File Name: Supplementary Data 1
Topologies obtained from three types of gene families, namely families for which (i) R. chiliantha and other Juglandaceae species have two gene copies; (ii) R. chiliantha has two gene copies while the other Juglandaceae species has one copy; and (iii) R. chiliantha has one gene copy while the other Juglandaceae species has two copies. aBS: Ultrafast bootstrap (UFBoot) support (%) value.

File Name: Supplementary Data 2
Description: Annotation result of DNA repair and recombination genes in seven Juglandaceae species.

File Name: Supplementary Data 3
Description: Results of the orthogroup analysis for DNA repair and recombination genes in seven Juglandaceae species.

File Name: Supplementary Data 4
Description: RNA-seq data for 54 DNA repair and recombination genes in two tissues (leaf and flower bud) of *Rhoiptelea chiliantha.*

File Name: Supplementary Data 5
Description: Numbers of ancestral genes on homoeologous chromosome pairs.

File Name: Supplementary Data 6
Description: Information on the Juglandaceae subgenomes assigned by intraspecific collinear blocks.

File Name: Supplementary Data 7
Description: Results from MSCquartet analysis when the two subgenomes were assigned by homoeologous chromosomes.

File Name: Supplementary Data 8
Description: Results from MSCquartets analysis when the two subgenomes were assigned by intraspecific collinear blocks.

File Name: Supplementary Data 9
Description: Gene function and duplication type for BUSCO genes classified as multi-copy genes in *Rhoiptelea chiliantha*.
